# Genetic Manipulation of Competition for Nitrate between Heterotrophic Bacteria and Diatoms

**DOI:** 10.3389/fmicb.2016.00880

**Published:** 2016-06-09

**Authors:** Rachel E. Diner, Sarah M. Schwenck, John P. McCrow, Hong Zheng, Andrew E. Allen

**Affiliations:** ^1^Integrative Oceanography Division, Scripps Institution of Oceanography, University of California San DiegoLa Jolla, CA, USA; ^2^Microbial and Environmental Genomics Group, J. Craig Venter InstituteLa Jolla, CA, USA

**Keywords:** diatoms, bacteria, nitrate, competition, genetic manipulation, transcriptomics, *Phaeodactylum*, *Alteromonas*

## Abstract

Diatoms are a dominant group of eukaryotic phytoplankton that contribute substantially to global primary production and the cycling of important elements such as carbon and nitrogen. Heterotrophic bacteria, including members of the gammaproteobacteria, are commonly associated with diatom populations and may rely on them for organic carbon while potentially competing with them for other essential nutrients. Considering that bacterioplankton drive oceanic release of *CO*_2_ (i.e., bacterial respiration) while diatoms drive ocean carbon sequestration vial the biological pump, the outcome of such competition could influence the direction and magnitude of carbon flux in the upper ocean. Nitrate availability is commonly a determining factor for the growth of diatom populations, particularly in coastal and upwelling regions. Diatoms as well as many bacterial species can utilize nitrate, however the ability of bacteria to compete for nitrate may be hindered by carbon limitation. Here we have developed a genetically tractable model system using the pennate diatom *Phaeodactylum tricornutum* and the widespread heterotrophic bacteria *Alteromonas macleodii* to examine carbon-nitrogen dynamics. While subsisting solely on *P. tricornutum* derived carbon, *A. macleodii* does not appear to be an effective competitor for nitrate, and may in fact benefit the diatom; particularly in stationary phase. However, allochthonous dissolved organic carbon addition in the form of pyruvate triggers *A. macleodii* proliferation and nitrate uptake, leading to reduced *P. tricornutum* growth. Nitrate reductase deficient mutants of *A. macleodii* (Δ*nasA*) do not exhibit such explosive growth and associated competitive ability in response to allochthonous carbon when nitrate is the sole nitrogen source, but could survive by utilizing solely *P. tricornutum*-derived nitrogen. Furthermore, allocthonous carbon addition enables wild-type *A. macleodii* to rescue nitrate reductase deficient *P. tricornutum* populations from nitrogen starvation, and RNA-seq transcriptomic evidence supports nitrogen-based interactions between diatoms and bacteria at the molecular level. This study provides key insights into the roles of carbon and nitrogen in phytoplankton-bacteria dynamics and lays the foundation for developing a mechanistic understanding of these interactions using co-culturing and genetic manipulation.

## Introduction

Diatoms as well as bacteria are important drivers of oceanic biogeochemical cycling, and frequently occupy overlapping ecological niches. Diatoms are often the dominant primary producers in nutrient rich ecosystems, such as coastal upwelling regions, and can form dense and extensive blooms. Marine bacteria constitute the majority of oceanic biomass (Pomeroy, 1974; Fuhrman, 1989; Whitman et al., 1998), and heterotrophic bacteria utilize and rely on phytoplankton-derived organic matter for survival and growth (Azam and Malfatti, 2007; Sarmento and Gasol, 2012; Buchan et al., 2014). Diatoms and bacteria are subject to frequent environmental fluctuations in availability of essential nutrients such as nitrogen (N) and carbon (C). Nitrate (${\text{NO}}_{3}^{-}$) in particular often reaches limiting concentrations during phytoplankton blooms (Falkowski and Oliver, 2007). Certain classes of heterotrophic bacteria, such as gammaproteobacteria, are consistently found in phytoplankton-associated microbial communities, and may potentially compete with diatoms for scarce nutrients while simultaneously relying on them for organic C (Buchan et al., 2014). While population dynamics of phytoplankton and bacteria under different environmental conditions have been extensively examined, outside of a few recent studies (e.g., Durham et al., 2014; Amin et al., 2015; Smriga et al., 2016) relatively little is known regarding the cellular, metabolic, or genetic basis for different types of interactions (Bell and Mitchell, 1972; Amin et al., 2012). This is particularly true for competitive interactions (Amin et al., 2012). Laboratory model systems and new experimental approaches can enable hypothesis-testing and lead to new discoveries regarding interactions between diatoms and heterotrophic bacteria in productive microbial ecosystems and the associated influence on C and nutrient cycling.

A common regulator of primary productivity in marine ecosystems is availability of ${\text{NO}}_{3}^{-}$. When estimating primary productivity and characterizing the magnitude of the biological pump it is assumed that inorganic N is converted into particulate organic matter entirely by phytoplankton (Dugdale and Goering, 1967; Bronk et al., 1994). Diatoms are excellent competitors for ${\text{NO}}_{3}^{-}$, and have evolved efficient assimilation, storage and associated recycling systems (Serra et al., 1978; Dortch, 1990; Lomas and Gilbert, 2000; Allen et al., 2006, 2011). The emerging laboratory model diatom *Phaeodactylum tricornutum* has been used in many studies to investigate diatom N utilization, as well as responses to many other environmental variables such as iron (Fe) and phosphorus (P) (Yongmanitchai and Ward, 1991; Geider et al., 1993; Allen et al., 2008; Jiao et al., 2011; Matthijs et al., 2015; Morrissey et al., 2015). These studies have been facilitated by development of a variety of tools for genetic manipulation in *P. tricornutum* (Siaut et al., 2007; Karas et al., 2015; Weyman et al., 2015; Nymark et al., 2016). Notably, mutant strains of *P. tricornutum* that are deficient in ability to utilize ${\text{NO}}_{3}^{-}$ have been crucial for understanding N uptake and storage, and impacts on cellular physiology (Levitan et al., 2014). The sequencing of the *P. tricornutum* genome (Bowler et al., 2008) revealed that about 6% of *P. tricornutum* genes appear to be bacterial in origin, including a NAD(P)H dependent assimilatory ${\text{NO}}_{2}^{-}$, reductase, and are possibly the result of horizontal gene transfer. This suggests a historically intimate relationship between diatoms and bacteria, which might also have significant evolutionary implications.

Heterotrophic bacteria also play a large role in N cycling and remineralization (Zehr and Ward, 2002; Azam and Malfatti, 2007). They are known to utilize a variety of sources for satisfying their N requirements, including ammonium (${\text{NH}}_{4}^{+}$), ${\text{NO}}_{3}^{-}$, urea, free amino acids, and various other organic N compounds. In some studies, ${\text{NH}}_{4}^{+}$ and organic N sources such as amino acids have been shown to satisfy the bulk of heterotrophic bacterial N demand, while other organic N sources and inorganic N such as ${\text{NO}}_{3}^{-}$ appeared to play a more minor role (Wheeler and Kirchman, 1986; Keil and Kirchman, 1991). However, a large number of heterotrophic bacteria possess pathways for utilizing ${\text{NO}}_{3}^{-}$ and are able to grow using ${\text{NO}}_{3}^{-}$ as a sole N source. Studies examining the molecular ecology of heterotrophic bacterial nitrate reductase genes (*nasA*) and their functionality have suggested that bacterial ${\text{NO}}_{3}^{-}$ utilization is globally widespread and may play an important role in inorganic N cycling in several ecosystems (Allen et al., 2001; Jiang et al., 2015). Further, heterotrophic bacteria have been shown to satisfy between 10 and 50% of their total N demand with dissolved inorganic nitrogen (${\text{NH}}_{4}^{+}$ and ${\text{NO}}_{3}^{-}$), and can account for between 10 and 40% of total water column ${\text{NO}}_{3}^{-}$ uptake (Allen et al., 2005). Stable isotope probing (SIP) experiments with ^15^${\text{NO}}_{3}^{-}$ have also shown that heterotrophic bacteria in natural assemblages, including members of the *Alteromonas* genera, can and do take up ${\text{NO}}_{3}^{-}$ (Wawrik et al., 2012). Methods based on sorting heterotrophic and autotrophic cells with flow cytometry following ^15^${\text{NO}}_{3}^{-}$ incubation have also documented significant levels of heterotrophic bacterial ${\text{NO}}_{3}^{-}$ utilization (Bradley et al., 2010a,b,c; Lomas et al., 2011). However, the role that bacterial ${\text{NO}}_{3}^{-}$ assimilation plays in shaping microbial communities and regulating ${\text{NO}}_{3}^{-}$ flux in pelagic ecosystems remains poorly understood, though it may have important consequences for understanding the biological pump.

To gain a deeper understanding of N-related interactions between diatoms and bacteria, we developed a model co-culture system using the diatom *P. tricornutum* CCMP 632 and the marine heterotrophic bacteria *Alteromonas macleodii. A. macleodii* represents an excellent model for investigating these interactions because *Alteromonas* sp. are ecologically important members of the gammaproteobacteria class, and have been shown to be amenable to genetic manipulation (Kato et al., 1998; Weyman et al., 2011). They are relatively large (~1-2 μm), rod-shaped motile bacteria that are capable of utilizing a variety of C and N sources (López-Pérez and Rodríguez-Valera, 2014; Pedler Sherwood et al., 2015). Ecologically, they are frequently associated with nutrient and particle-rich environments and are commonly found as active and dominant members of phytoplankton-associated bacterial assemblages (Buchan et al., 2014; López-Pérez and Rodríguez-Valera, 2014). *Alteromonas* bacteria have previously been shown to interact with individual eukaryotic and prokaryotic phytoplankton species. These interactions range from impairing algal growth, sometimes by algicidal means (Kato et al., 1998; Mayali and Azam, 2004; Aharonovich and Sher, 2016) to effects that are either neutral or beneficial to algal growth in co-culture (Morris et al., 2008, 2011; Sher et al., 2011; Aharonovich and Sher, 2016). When concentrated organic matter is available, *Alteromonas* bacteria have been shown to be among the most rapidly dividing heterotrophic prokaryotes, and can reach high population densities (Shi et al., 2012; Pedler et al., 2014). *A. macleodii*, the designated type species for the *Alteromonas* genus, is distributed globally and is exceptionally diverse genetically (García-Martínez et al., 2002; Ivars-Martínez et al., 2008; López-Pérez et al., 2012). The strain selected for this study, *A. macleodii* ATCC27126, is capable of utilizing ${\text{NO}}_{3}^{-}$ as a sole N source in minimal (Aquil) media supplemented with a dissolved organic carbon (DOC) source, solidifying this strain as an excellent candidate for the model system employed in this study.

Through the use of targeted genetic manipulation we have gained new insights into the mechanisms governing physiological processes related to nutrient exchange and competition between diatoms and bacteria, particularly interactions involving C and N. We examined model diatoms and bacteria that are both capable of ${\text{NO}}_{3}^{-}$ utilization. Leveraging new and existing genetic tools available for each organism in the model system presented here, we created both diatom and bacterial mutants lacking the ability to utilize ${\text{NO}}_{3}^{-}$. We then examined the response of these strains in co-culture under varying C and N availability scenarios. Additionally, we conducted transcriptional profiling experiments in order to identify molecular responses of diatoms to the bacteria as well as to gain insights into the physiological status of each partner.

## Materials and methods

### Strains and culturing conditions

Axenic cultures of *P. tricornutum* strain CCMP 632 were obtained from the Provasoli-Guillard National Center for Culture of Marine Algae and Microbiota. Axenic cultures were confirmed via microscopy (light and DAPI staining), in addition to regular plating on marine bacterial growth media. *P. tricornutum* monocultures and *A. macleodii* co-cultures were grown in Aquil artificial seawater media (ASW), with modified concentrations of ${\text{NO}}_{3}^{-}$ added as sodium nitrate (Fisher Bioreagents, Waltham MA, USA) or ${\text{NH}}_{4}^{+}$ added as ammonium chloride (Fisher Bioreagents, Waltham MA, USA). Media was microwaved to ~95°C two times prior to cooling, addition of nutrients and filter sterilization (0.2 μm bottle-top filters, Thermo Fisher Scientific, Waltham MA, USA). Experiments were conducted at 18°C with a light intensity of 170 μmol photons m^−2^ s^−1^ and a 14/10 h light/dark cycle. In order to minimize variability resulting from diel effects, measurements and sampling for each experiment occurred at the same time each day, ~6 h after light cycle onset.

*A. macleodii* strain ATCC27126 monocultures were grown routinely either on Zobell 2216 marine broth (MB) 1% agar plates, or in MB liquid medium at 28°C. *A. macleodii* growth was also supported in half-strength marine broth media, and in Aquil ASW supplemented with 5 mM pyruvate. Liquid cultures were grown shaking at 225 rpm. Prior to co-culturing experiments, overnight cultures of *A. macleodii* culture were centrifuged at 6000 x g for 3 min; the supernatant was discarded, and cells were subsequently gently washed 3 times in the experimental seawater media. *E. coli* used for cloning was cultured in LB broth (Amresco, Solon OH, USA) or on LB agar plates at 37°C. Antibiotic concentrations for selective bacterial growth were provided as 100 μg/ml Kanamycin and 100 μg/ml Ampicillin for *A. macleodii* and 50 μg/ml Kanamycin, 100 μg/ml Ampicillin, 10 μg/ml Chloramphenicol, 10 μg/ml Tetracycline, or 10 μg/ml Spectinomycin for *E. coli* as needed.

### Genetic manipulation of *A. macleodii* and *P. tricornutum*

Previous studies genetically manipulating *Alteromonas* bacteria focused on either undesignated species (Kato et al., 1998) or species other than *A. macleodii* (Weyman et al., 2011 focused on the “deep ecotype” which was reclassified as *A. mediterranea*), making this study the first to genetically manipulate this widespread species. The genome sequence of *A. macleodii* strain ATCC27126 was obtained from the JGI IMG data base and the DNA sequence of the single copy nasA gene was identified and used to design the knockout (KO) construct. The *A. macleodii* Δ*nasA* line was engineered using SacB-mediated homologous recombination, as in Weyman et al. (2011). Gibson assembly was used to construct the suicide plasmid pRED16 (Supplementary Figure 1A), which contains an origin of replication from source plasmid pBBR1-MCS5 (incapable of replication in *A. macleodii* ATCC27126), an origin of transfer, a SacB gene conferring toxicity to sucrose, and 2 1-kb regions homologous to the *A. macleodii nasA* gene flanking a kanamycin resistance cassette (Kovach et al., 1995; Gibson et al., 2009; Weyman et al., 2011). This plasmid was assembled and transformed into *E. coli*, which was mated overnight with the *A. macleodii* WT strain. Transconjugants were dilution-plated to obtain Kanamycin resistant single colonies, and then streaked onto 5% sucrose plates to select for double-crossover recombinants. These were again plated to single colonies, which were screened by PCR to amplify regions specific to *A. macleodii* strain ATCC27126 (to confirm sole presence of this strain as the primers do not amplify in *E. coli*), and regions spanning both junctions of the genome insert, as well as the entire insert (data not shown). All colonies screened were identified as *A. macleodii* strain ATCC27126 and were positive for the KO insert. The KO phenotype (inability to utilize ${\text{NO}}_{3}^{-}$) was confirmed by plating transconjugant colonies and the WT strain on seawater-agar plates containing pyruvate as a C source, either ${\text{NO}}_{3}^{-}$ or ${\text{NH}}_{4}^{+}$ as a N source, and X-gal solution (Takara, Meadow View CA, USA) to better visualize the phenotypic effect. The WT strain was able to grow on either N source, while the knockout strain displayed growth on ${\text{NH}}_{4}^{+}$ but not on ${\text{NO}}_{3}^{-}$ (Supplementary Figure 1B).

*P. tricornutum* nitrate reductase knockout (NRKO) lines were constructed using Transcription activator-like effector nuclease (TALEN) genetic manipulation, as in Weyman et al. (2015). Using the JGI *P. tricornutum* genome (http://genome.jgi.doe.gov/Phatr2/Phatr2.home.html), the sequence encoding nitrate reductase (NR) (Phatr2 ID: 54983, Phatr3 ID: J54983) was identified and activity was eliminated by interrupting the sequence with a phleomycin-resistance cassette suitable for downstream selection. Transformation of the NRKO plasmids was accomplished by microparticle bombardment (BIO-RAD PDS-1000/He Biolistic Particle Delivery System).

### Experimental design

**Figure 1:**
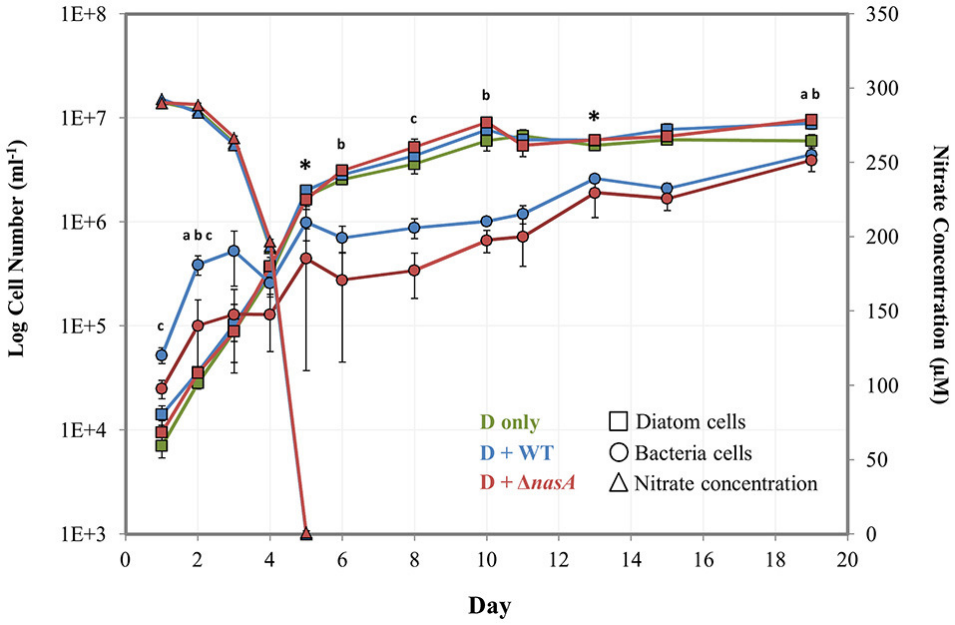
**Log number of bacteria and diatom cells (left vertical axis) determined by flow cytometry and nitrate concentrations (right vertical axis) determined by spectrophotometry during the baseline experiment**. Squares = diatom cell numbers, circles = bacterial cell numbers, and triangles = nitrate concentrations (μM). Green markers = *P. tricornutum* monocultures (also noted as D only), blue markers = *P. tricornutum*–*A. macleodii* WT co-cultures (also noted as D + WT), and red markers = *P. tricornutum*–*A. macleodii* Δ*nasA* co-cultures (also noted as D + Δ*nasA*). Exponential growth stage was defined as days 1–6, and stationary growth phase as days 7–19. ^*^ indicates the two sampling points for physiology and transcriptomics. ^*a*^ indicates that diatom cell numbers were significantly different between *P. tricornutum* monocultures and *A. macleodii* WT co-cultures. ^*b*^ indicates that diatom cell numbers were significantly different between *P. tricornutum* monocultures and *A. macleodii* Δ*nasA* co-cultures. ^*c*^ indicates that bacteria cell numbers were significantly different between the WT and Δ*nasA* co-cultures. All data points represent the average of *n* = 3 replicates, and error bars are standard deviation. Some differences in cell numbers and nitrate concentrations (e.g., diatom monoculture nitrate concentrations, which are overlapped by the Δ*nasA* co-culture measurements) are difficult to discern, so average non-transformed cell numbers and nitrate concentrations have been listed in Supplementary Table 2.

To address baseline physiological and transcriptional profiles of diatom and diatom-bacteria co-cultures, 1L batch cultures were grown in autoclaved 2L Erlenmeyer flasks. Three treatments with three biological replicates each were examined: *P. tricornutum* monocultures, *P. tricornutum*–*A. macleodii* WT co-cultures, and *P. tricornutum*–*A. macleodii* Δ*nasA* co-cultures. Prior to starting the experiments, both phytoplankton and bacteria were cultured together in the relevant experimental conditions for >7 *P. tricornutum* generations (~1 week), and inoculated during *P. tricornutum* exponential phase. Sampling was conducted daily for spectrophotometric measurement of ${\text{NO}}_{3}^{-}$, and for cell counts via flow cytometry (see below). Samples for additional physiological parameters and RNA-seq transcriptomic analysis were also collected during *P. tricornutum* exponential and stationary phase (Figure 1). Physiological parameters included *in vivo* fluorescence, pH, Chl-a, Fv/Fm, and dissolved and particulate organic C and N. The initial ${\text{NO}}_{3}^{-}$ concentration was 300 μM. Diatom specific growth rates (μ) were calculated using cell densities obtained via flow cytometry on day 2 and day 5 of the experiment. For comparison to flow cytometry results, *P. tricornutum* cells were also counted on a counting chamber and bacteria colony forming units (CFU) ml^−1^ were determined for the *A. macleodii* (see below). Every second day, the *P. tricornutum* monocultures were plated on MB and co-cultures were plated on MB with Kanamycin in order to confirm lack of culture cross-contamination.

Several small-scale experiments were performed to examine the effects of C and N concentration on co-culture population dynamics, interactions between bacteria and the *P. tricornutum* NRKO line, and *A. macleodii* growth on multiple media types. For these experiments, 20ml cultures and co-cultures were grown in sterile glass tubes in triplicate and 300 μL samples were collected regularly and preserved with paraformaldehyde (PFA) for subsequent processing via flow cytometry (see below). Four hundred microlitre samples were also collected in order to measure ${\text{NO}}_{3}^{-}$ concentration in the DOC addition experiment (see below). *A. macleodii* growth on different media sources was evaluated by growing overnight cultures of *A. macleodii* WT, gently rinsing the cells two times in nutrient free Aquil ASW, and resuspending in 1 ml of the same media prior to inoculating media treatments with 1:1000 dilution of the bacteria. Cells were grown in the following media treatments: Aquil ASW with and without 300 μM ${\text{NO}}_{3}^{-}$ (Aq and Aq-N, respectively), MB, and expired media from a *P. tricornutum* stationary culture filtered through a 0.2 μm filter (PtF).

In DOC addition experiments, co-cultures that had been maintained semi-continuously were used for inoculations. For both the *A. macleodii* WT and Δ*nasA*-*P. tricornutum* co-cultures, five different DOC-addition treatments were established (each in triplicate): DOC added either at the time of inoculation (Day 0) or on days 2, 4, 6, and 8 of the experiment. A no DOC-addition control was included for a total of 6 treatments. Allochthonous DOC was added as pyruvate at a concentration of 5 mM. Media contained 300 μM final concentration of ${\text{NO}}_{3}^{-}$. As a result of pyruvate interference with spectrophotometer measurement of ${\text{NO}}_{3}^{-}$ samples were sent for autoanalyzer ${\text{NO}}_{3}^{-}$ analysis (see below).

To address the impacts of varying C and ${\text{NO}}_{3}^{-}$ concentrations on *P. tricornutum* and *A. macleodii* WT population dynamics, co-cultures maintained semi-continuously from the baseline experiment were used to inoculate experiments using nine different media types. ${\text{NO}}_{3}^{-}$ concentrations of 50 μM, 300 μM, and 1 mM and DOC concentrations of 0 μM, 50 μM, and 1 mM were tested in a factorial design. Twenty milliliter co-cultures were grown in sterile glass tubes in triplicate and 500 μL samples were collected for flow cytometry analysis on days 2, 4, 6, and 31. Co-cultures containing 300 μM ${\text{NO}}_{3}^{-}$ and no DOC addition were cultured semi-continuously for 5 subsequent transfers to determine steady-state growth rates of *P. tricornutum* in co-culture. To account for variability in cell concentration, μ was determined using the highest growth rate observed between any 2 subsequent sampling points in each transfer experiment.

For the *P. tricornutum* NRKO co-culturing experiments, three different co-cultures were grown in both ${\text{NH}}_{4}^{+}$ and ${\text{NO}}_{3}^{-}$ amended Aquil ASW; *P. tricornutum* NRKO only, *P. tricornutum* NRKO + WT *A. macleodii*, and *P. tricornutum* NRKO + WT *A. macleodii* + DOC. The *P. tricornutum* NRKO with and without *A. macleodii* were acclimated in ${\text{NH}}_{4}^{+}$ amended media after which they were inoculated into both media types and pyruvate was added to the relevant treatments. N was added as 880 μM final concentration of the relevant source. Since some of the experiments included DOC amendments, we conducted an experiment to examine the impact of such DOC addition on *P. tricornutum* growth. *P. tricornutum* WT monocultures and *P. tricornutum* WT monocultures + 5 mM pyruvate cultures were established in 20 ml cultures as above, and run in triplicate, and media contained 880 μM final concentration of ${\text{NO}}_{3}^{-}$.

### Sample collection and processing: Cell numbers, physiology, and ${\text{NO}}_{3}^{-}$ drawdown

Cell densities of diatoms and bacteria were determined by flow cytometry, and in the case of the baseline experiment, also by manual counting methods. *P. tricornutum* cells were counted manually on either a Sedgwick-Rafter hemocytometer (when cell densities were low) or an Improved-Neubauer hemocytometer (IN-Cyto, Chungnam-do, Korea). *A. macleodii* CFU were determined by dilution-plating onto MB agar plates. (Both WT and Δ*nasA* lines of *A. macleodii* were also plated on MB kanamycin plates to confirm the sole presence of Δ*nasA* lines. Samples for staining and cell counting via flow cytometry were preserved by adding PFA to a final concentration of 0.5%. Samples were incubated at 4°C prior to flash freezing and storage at −80°C until processing. Flow cytometry analysis was conducted on a BD FACS Aria II using the Bacteria Kit for flow cytometry (Thermo Fisher Scientific, Waltham MA, USA) for quantifying the number of diatom and bacteria cells in each sample. After the addition of beads to samples, SYBR green I DNA stain was added, effectively staining all three populations (diatoms, bacteria, and beads). Bacterial and diatom populations were quantified simultaneously, and typical flow cytometer settings were forward scatter (FSC) = 200, side scatter (SSC) = 250, FSC PMT = 550, SYBR Green (SYBR Grn) = 530, Yellow fluorescence (YFP) = 335, with the following thresholds: SSC = 200, FSC = 200.

Nitrate levels were measured either via spectrophotometer or autoanalyzer (in DOC addition experiments). For spectrophotometric measurement, samples were prepared by centrifuging whole sample in Eppendorf tubes at 8000g for 10 min. Supernatant was then recovered, and stored at −20°C until processing. ${\text{NO}}_{3}^{-}$ values were determined by generating a standard curve using dilutions of 880 μM ${\text{NO}}_{3}^{-}$ media, using only curves with >99% precision (Collos et al., 1999; Johnson and Coletti, 2002). Samples were then measured in triplicate. As allochthonous DOC addition interfered with the spectrophotometric readings, ${\text{NO}}_{3}^{-}$ and ${\text{NO}}_{2}^{-}$ analysis for DOC addition experiments was conducted using a Lachat QuikChem 8500 autoanalyzer as in Parsons et al. (1984).

Concentrations of total and dissolved organic C and N were determined using a Total Organic Carbon (TOCL) analyzer (Shimadzu) paired with an ASI-L autosampler (Shimadzu). Prior to sample collection, 24-ml glass vials were combusted at 450°C and vial caps were acid rinsed for >24 h. Five to ten milliliters samples were stored at −20°C prior to dilution with milliQ and processing. Standard curves for determining C and N concentrations were generated using automated dilution and sampling of 1000 ppm potassium hydrogen phthalate for C, and 1000 ppm potassium nitrate for N. Samples for determining C and N in the dissolved fraction were collected by gentle filtration through a 0.2 μm syringe filter. Particulate values were determined by subtracting dissolved values from total values. Chlorophyll A (Chl a) concentrations were measured by 90% acetone extraction. Ten milliliters of the sample was gently filtered onto GF/F filters, flash frozen in in liquid N_2_, and stored at −20°C before overnight acetone extraction and measurement on a 10AU fluorometer (Turner). Culture Fv/Fm was measured using a pam-fluorometer (WALZ). pH was measured using an InLab Expert pH probe (Mettler Toledo), calibrated using 4, 7, and 10 pH standards (Orion Application Solutions).

### Sample collection and processing: Transcriptomics

Transcriptomics samples were collected by gentle filtration onto 47 mm 0.2um polycarbonate filters (Whatmann), followed by flash freezing in liquid N_2_ and storage at −80°C prior to processing. Total RNA was isolated using Trizol reagent (Thermo Fisher Scientific, Waltham MA, USA). The TURBO DNA-free Kit (Thermo Fisher Scientific, Waltham MA, USA) was used to digest genomic DNA. RNA was subsequently purified further using the Agencourt RNAClean XP kit (Beckman Coulter, Carlsbad CA, USA). The quality of RNA was evaluated using an Agilent 2100 Bioanalyzer (also used for subsequent quality analyses). Ribosomal RNA was removed using Ribo-Zero Magnetic kits (Epicentre, San Diego CA, USA) with a modification of the removal solution, using a mixture of the plant, bacterial, and human/mouse/rat Removal Solutions in a ratio of 2:1:1. Following mRNA enrichment via rRNA removal, RNA quality was further inspected via bioanalyzer. The Ovation RNA-Seq System V2 (NuGEN Technologies, Inc.) was used for first and second strand cDNA synthesis and amplification, followed by evaluation of cDNA quality via bioanalyzer. cDNA was sheared using the S2/E210 focused-ultrasonicator (Covaris) with a target size of 300 bp, confirmed by bioanalyzer. Libraries for sequencing were constructed using the Ovation® Ultralow System V2 (NuGEN Technologies, Inc.), and the quality of libraries verified by bioanalyzer prior to sequencing. Libraries were quantified using qPCR and a Library Quantification Kit (Kapa Biosystems), prior to sequencing on an Illumina NextSEQ500 DNA sequencer.

Paired Illumina reads were filtered for Illumina primer contamination and quality trimmed to Phred score 33 and a minimum length of 30 prior to read mapping. Reads were mapped to target genome contigs of *P. tricornutum* (http://genome.jgi.doe.gov/Phatr2/Phatr2.home.html) and *A. macleodii* ATCC 27126 using BWA MEM alignment (Li, 2013). Raw read counts were calculated for each gene using featureCounts (Liao et al., 2014) based on gene models for *A. macleodii* (CP003841) and *P. tricornutum* (Phatr3, http://protists.ensembl.org/Phaeodactylum_tricornutum/Info/Index). Additional gene level *de novo* functional annotation was generated for *P. tricornutum* via KEGG, KO, KOG, Pfam, and TIGRfam assignments. RPKMs were computed using library mapped reads and lengths of CDS for each gene. Biological triplets were used to quantitatively estimate differential expression using edgeR (Robinson et al., 2010) to assign normalized fold-change and Benjamini-Hochberg adjusted *p*-values for each gene. Raw read counts for each gene were used in all edgeR analyses. Sequencing data generated as part of this study has been deposited at NCBI.

### Statistical analyses

In the baseline experiment, statistical analyses were conducted to examine potential differences in diatom cell numbers between the monoculture and co-culture treatments, bacteria cell numbers between co-culture treatments, and to explore whether any physiological parameters were different between treatments during the exponential or stationary phase. One-way ANOVAs were performed followed by Tukey HSD *post-hoc* tests. Statistical significance was assumed at *p* ≤ 0.05. All statistical analyses were conducted using R (version 2.14.2), and were performed on raw data (i.e., not transformed).

## Results

### Baseline physiology and transcriptomics in diatom-bacteria co-cultures

A baseline experiment was conducted initially to examine cell growth, culture physiology, N drawdown, and diatom gene expression in diatom-bacteria co-cultures. In this experiment, N was provided as ${\text{NO}}_{3}^{-}$ and no DOC was added. Cell counts obtained via flow cytometry were used to examine population dynamics of *P. tricornutum* and the *A. macleodii* strains in co-culture, which were compared to manual cell counts. Diatom growth stages for this experiment were defined generally as exponential (Day 1–5) and stationary (Day 6–19). The presence of WT or Δ*nasA A. macleodii* in *P. tricornutum* cultures did not significantly affect growth rate (Supplementary Table 1). Maximum diatom cell densities were typically similar among treatments during diatom exponential phase, but were higher in bacteria-containing cultures during stationary phase (Figure 1, Supplementary Table 2). These differences were significant between *P. tricornutum* monocultures and the *A. macleodii* Δ*nasA* co-cultures on day 2 (*p* < 0.01), day 6 (*p* < 0.05), day 10 (*p* < 0.05), and day 19 (*p* < 0.01), and between *P. tricornutum* monocultures and the *A. macleodii* WT line on day 1 (*p* < 0.05), day 2 (*p* < 0.005), and day 19 (*p* < 0.05; Figure 1). Although the maximum *P. tricornutum* cell densities estimated with manual counts were slightly lower than those calculated via flow cytometry, cell counts obtained from the two methods were similar (Supplementary Figure 2).

Both *A. macleodii* strains were able to grow in co-culture with *P. tricornutum*, as indicated by a gradual increase in cell abundance following *P. tricornutum* exponential stage (Figure 1). This growth increase did not occur when *A. macleodii* was grown in Aquil ASW in the absence of *P. tricornutum* (Supplementary Figure 3). The *A. macleodii* WT strain maintained higher numbers than the Δ*nasA* line, though patterns of growth were similar for both strains (Figure 1). These differences were significant (*p* < 0.05) on days 1, 2, and 8 of the experiment. After an initial increase in bacterial cell number in both bacteria co-culture treatments, bacterial numbers either declined (WT) or plateaued (Δ*nasA*) during the *P. tricornutum* exponential phase (Figure 1). Subsequently, cell numbers of both strains increased during the diatom stationary phase beginning on day 5. Manual CFU counts of the bacteria showed generally the same pattern of growth, however the growth decrease during *P. tricornutum* exponential phase was more dramatic and overall CFU ml^−1^ were lower following this phase in the experiment (Supplementary Figure 2). This difference in cell counts due to methodology has been observed in prior studies (Singleton et al., 1982; Mouriño-Pérez et al., 2003), where CFU counts were lower than direct counts under low DOC conditions. Likely the CFU counts reflect only viable, culturable cells while flow cytometry counts represent all cells including dormant, active, and recently dead cells. Neither *A. macleodii* strain was observed to physically attach to *P. tricornutum* at any point of the *P. tricornutum* growth cycle (qualitative observation, data not shown).

Nitrate concentrations decreased rapidly as *P. tricornutum* cell concentrations increased, and were undetectable by day 5 in all treatments (Figure 1). *P. tricornutum* cell numbers continued to increase exponentially even after the complete depletion of ${\text{NO}}_{3}^{-}$ in the media (Figure 1). ${\text{NO}}_{3}^{-}$ drawdown was similar among treatments with and without bacteria. Samples for cell physiology that were collected during the diatom exponential (Day 5) and stationary phase (Day 13), including Chl-a, pH, and Fv/Fm, showed no significant differences between treatments (Supplementary Table 3). Organic C and N were also evaluated for both the dissolved and the particulate culture fractions, and no significant differences between the treatments were observed (Supplementary Table 3).

### Baseline transcriptomic analysis

Whole-genome transcriptome analyses were conducted at exponential (Day 5) and stationary (Day 13) sampling points. In general, a very low percentage of sequenced and mapped reads were associated with the *A. macleodii* genome, with slightly more observed in the diatom stationary samples than in the exponential samples (Table 1). The large majority of sequenced reads (>98% in all co-culture treatments) mapped to the *P. tricornutum* genome (Table 1). As a result, analyses of *A. macleodii* gene expression are not included in this study. Genes were considered to be significantly differentially expressed (DE) when the adjusted *p* < 0.05, and only genes with differential expression of > 0.75 fold are discussed. All data reported in this paper are deposited in the NCBI sequence read archive (BioProject accession no. PRJNA319251; BioSample accession nos. SAMN04884450- SAMN04884467).

**Table 1:** **Sequencing data collected for transcriptomic sampling points, including the total number of raw reads, the total number of trimmed reads, the percentage of trimmed reads that mapped to either the a genome or a gene model belonging to ***P. tricornutum*** or ***A. macleodii***, and the percentage of the total mapped reads that corresponded to the ***A. macleodii*** and ***P. tricornutum*** genomes**.

	**Raw Reads**	**Trimmed Reads**	**% Reads mapped to a genome**	**% Reads mapped to a gene model**	**% Mapped reads: *A. macleodii***	**% Mapped reads: *P. tricornutum***
**EXPONENTIAL SAMPLING POINT**						
*P. tricornutum* Only	1.0E + 07	9.9E + 06	84.18	58.29	0.00	100.00
*P. tricornutum* + *A. macleodii* WT	7.7E + 06	7.6E + 06	80.74	35.78	0.21	99.79
*P. tricornutum* + *A. macleodii* Δ*nasA*	9.2E + 06	9.0E + 06	82.78	36.60	0.28	99.72
**TOTAL**	2.7E + 07	2.7E + 07				
**STATIONARY SAMPLING POINT**						
*P. tricornutum* Only	2.9E + 07	2.8E + 07	88.16	43.52	0.00	100.00
*P. tricornutum* + *A. macleodii* WT	3.7E + 07	3.6E + 07	88.61	43.96	1.70	98.30
*P. tricornutum* + *A. macleodii* Δ*nasA*	3.0E + 07	3.0E + 07	88.92	33.69	1.90	98.10
**TOTAL**	9.5E + 07	9.4E + 07				

**Figure 2:**
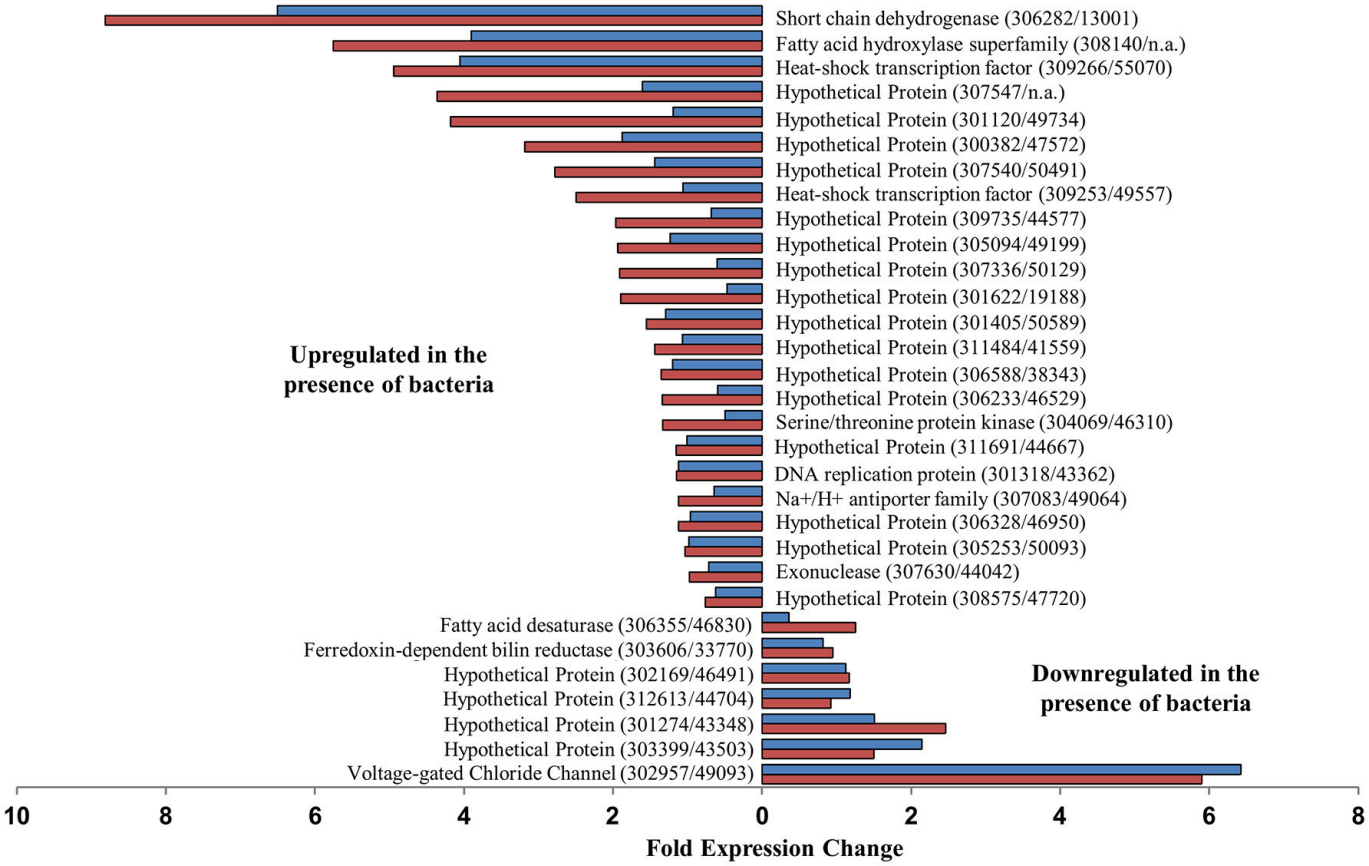
**Putative ***P. tricornutum*** genes that were significantly differentially expressed between ***P. tricornutum*** monocultures and both WT and Δ***nasA*** bacterial co-cultures, and fold expression change**. Blue bars = DE in WT co-cultures, red bars = DE in Δ*nasA* co-cultures. Gene descriptions are adjacent to relevant data bars, with gene IDs displayed as (Phatr3 ID/Phatr2 ID).

No *P. tricornutum* genes were significantly differentially expressed (DE) between any of the treatment during the diatom exponential sampling point, however, during the stationary sampling point many genes were differentially expressed between the *P. tricornutum* monoculture and either or both of the *P. tricornutum*-bacteria co-cultures. A set of 34 genes were significantly DE between axenic cultures and both bacterial co-cultures (Figure 2). The gene most highly upregulated in *P. tricornutum* in response to the bacteria (>5.9 fold in both co-cultures) is a putative voltage-gated ion channel (Phatr2 ID: 49093, Phatr3 ID: 302957) that has been shown to be involved in ${\text{NO}}_{3}^{-}$ sensing and transport (see discussion). Other upregulated genes include a putative ferredoxin-dependent bilin reductase (Phatr2 ID: 33770, Phatr3 ID: 303606), and a putative fatty acid desaturase (Phatr2 ID: 46830, Phatr3 ID: 306355). Several of the genes that were downregulated in *P. tricornutum* in response to bacteria were related to cellular information storage and processing such as transcriptional regulation and replication, including two different putative heat shock protein transcription factors. The two genes most highly downregulated in the presence of bacteria were a short chain dehydrogenase (Phatr2 ID: 13001, Phatr3 ID: 306282), which was downregulated 8.8 and 6.5-fold in the Δ*nasA* and WT co-cultures, respectively, and a fatty acid hydroxylase (Phatr2 ID: N/A, Phatr3 ID: 308140), which was downregulated 5.7 and 3.9-fold in the Δ*nasA* and WT co-cultures, respectively (Figure 2).

Several transcripts were either upregulated or downregulated in both co-cultures, but the difference was only significant for one of the co-cultures. An additional 9 genes were DE between *P. tricornutum* monocultures and the *P. tricornutum*–*A. macleodii* WT co-culture treatments (Supplementary Table 4). These include a putative ${\text{NO}}_{2}^{-}$ transporter (Phatr2 ID: 13076, Phatr3 ID: 308281), and a putative membrane associated ${\text{NO}}_{3}^{-}$ transporter (Phatr2 ID: 26029, Phatr3 ID: 307720), which exhibited a >2.5-fold difference. Both of these putative genes were upregulated in the *P. tricornutum* monoculture compared to the co-cultures, and had a larger expression change in the WT bacteria co-culture than the Δ*nasA* co-culture (Supplementary Table 4). There were 99 genes significantly DE in *P. tricornutum* monocultures compared to *P. tricornutum*-*A. macleodii* Δ*nasA* co-cultures (Supplementary Table 4), including upregulation of a putative glutamine synthetase gene (Phatr2 ID: 51092, Phatr3 ID: 306624), a putative tryptophan/tyrosine permease (Phatr2 ID: 45852, Phatr3 ID: 310088), and a putative ferredoxin nitrite reductase (Phatr2 ID: 12902, Phatr3 ID: 308097). A putative sugar transporter (Phatr3 ID: 49722, Phatr2 ID: 311238) was downregulated in diatom monocultures, as well as additional heat shock transcription factors, including one that was downregulated >19-fold (Phatr2 ID: 48554, Phatr3 ID: 304737).

We also identified and compared expression of genes related to ${\text{NH}}_{4}^{+}$ utilization and transport. 17 putative genes were identified (Supplementary Table 5), and none were significantly DE between any treatments at a given sampling point.

### Growth physiology of *A. macleodii* in multiple media types

In all media types, *A. macleodii* WT showed a rapid increase in cell number between inoculation on day 1 and the next measured time-point on day 3 (Supplementary Figure 3). Following this initial increase, cell numbers either decreased in all aquil-based media (Aq, Aq-N, and PtF media), or experienced a modest decrease followed by little change (MB media) (Supplementary Figure 3). The highest cell density occurred when cells were grown in MB media, reaching ~1 × 10^8^ cells ml^−1^. *A. macleodii* WT in PtF media reached a higher maximum cell density (2.2 × 10^6^ cells ml^−1^) than the Aq and Aq-N treatments (8.6 × 10^5^ and 8.9 × 10^5^ cells ml^−1^, respectively).

### Competition between diatoms and bacteria for ${\text{NO}}_{3}^{-}$

**Figure 3:**
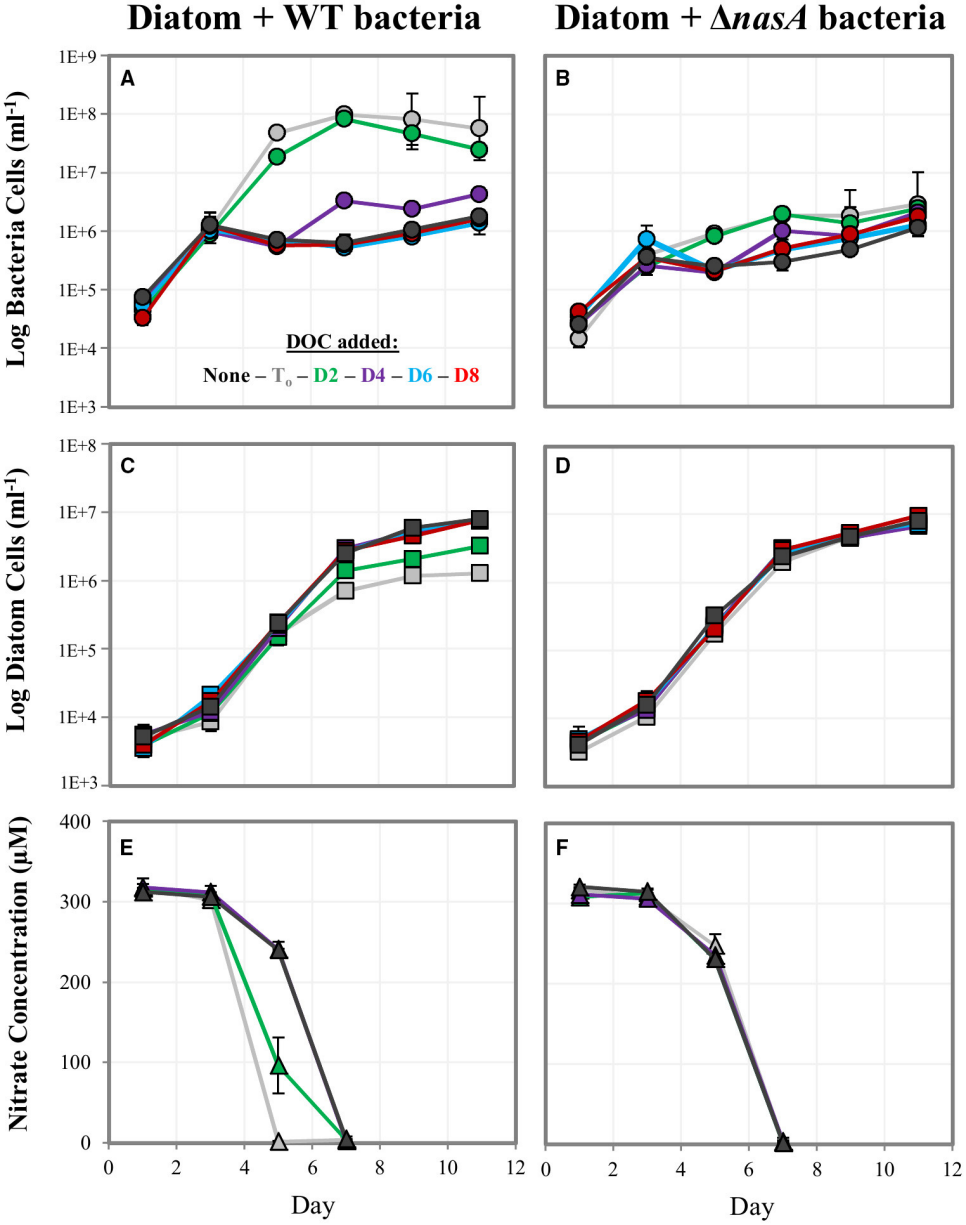
**Log cell numbers per ml and nitrate concentrations in DOC addition experiments**. **(A,C,E)** are co-cultures containing the WT *A. macleodii* strain, and **(B,D,F)** contain the *A. macleodii* Δ*nasA* strain. Colors represent the day of the experiment on which DOC was added: black = control, no DOC added, gray = day of inoculation (T_0_), green = day 2, purple = day 4, blue = day 6, red = day 8.

We examined whether bacteria could impede diatom growth by competing for ${\text{NO}}_{3}^{-}$ if sufficient DOC was present for bacterial growth. We hypothesized that the presence of DOC prior to ${\text{NO}}_{3}^{-}$ depletion could enable bacterial competition for ${\text{NO}}_{3}^{-}$, but that competition would not occur if (1) DOC is not sufficient for bacterial growth, or (2) bacteria are unable to utilize the ${\text{NO}}_{3}^{-}$ in the media. We tested this by culturing the diatoms with WT bacteria capable of utilizing ${\text{NO}}_{3}^{-}$ and Δ*nasA* bacteria unable to utilize ${\text{NO}}_{3}^{-}$ as a N source. We then added DOC at multiple time-points and included a no DOC addition control to better understand any competitive interactions observed, measuring diatom and bacteria growth and ${\text{NO}}_{3}^{-}$ in the media (indicative of biological ${\text{NO}}_{3}^{-}$ drawdown). In co-cultures containing Δ*nasA* bacteria, diatom growth and ${\text{NO}}_{3}^{-}$ drawdown were similar in all treatments regardless of whether and when DOC was added (Figures 3B,D,F). Numbers of Δ*nasA* bacteria increased slightly when DOC was added prior to the depletion of ${\text{NO}}_{3}^{-}$ from the media, but were not affected when DOC was added on days after ${\text{NO}}_{3}^{-}$ depletion. In co-cultures containing diatoms and WT bacteria, when DOC was added early in the experiment (Day T_o_ or Day 2), bacteria cell numbers increased dramatically and diatom cell numbers reached lower maximum cell densities (Figures 3A,C). Diatom cell numbers with DOC added on Day T_o_were > 6 times lower than in the no DOC addition control. Media ${\text{NO}}_{3}^{-}$ was also depleted earlier in these cultures (Figures 3E,F), indicating a relationship between bacterial growth, ${\text{NO}}_{3}^{-}$ drawdown, and diatom growth impairment. When DOC was added on later time points (Day 4, 6, and 8), diatom growth and ${\text{NO}}_{3}^{-}$ drawdown were similar to the no DOC addition control. Bacteria growth increased slightly when DOC was added on Day 4, but DOC addition on subsequent days had no effect.

*P. tricornutum* cultures with and without the addition of pyruvate (final concentration 5 mM, as used in other DOC addition experiments) did not differ significantly in cell numbers as measured at 3 different time points (Supplementary Figure 4). Cell numbers were determined on days 4, 7, and 15 of the experiment.

### Population dynamics of NRKO diatoms and WT bacteria in co-culture

**Figure 4:**
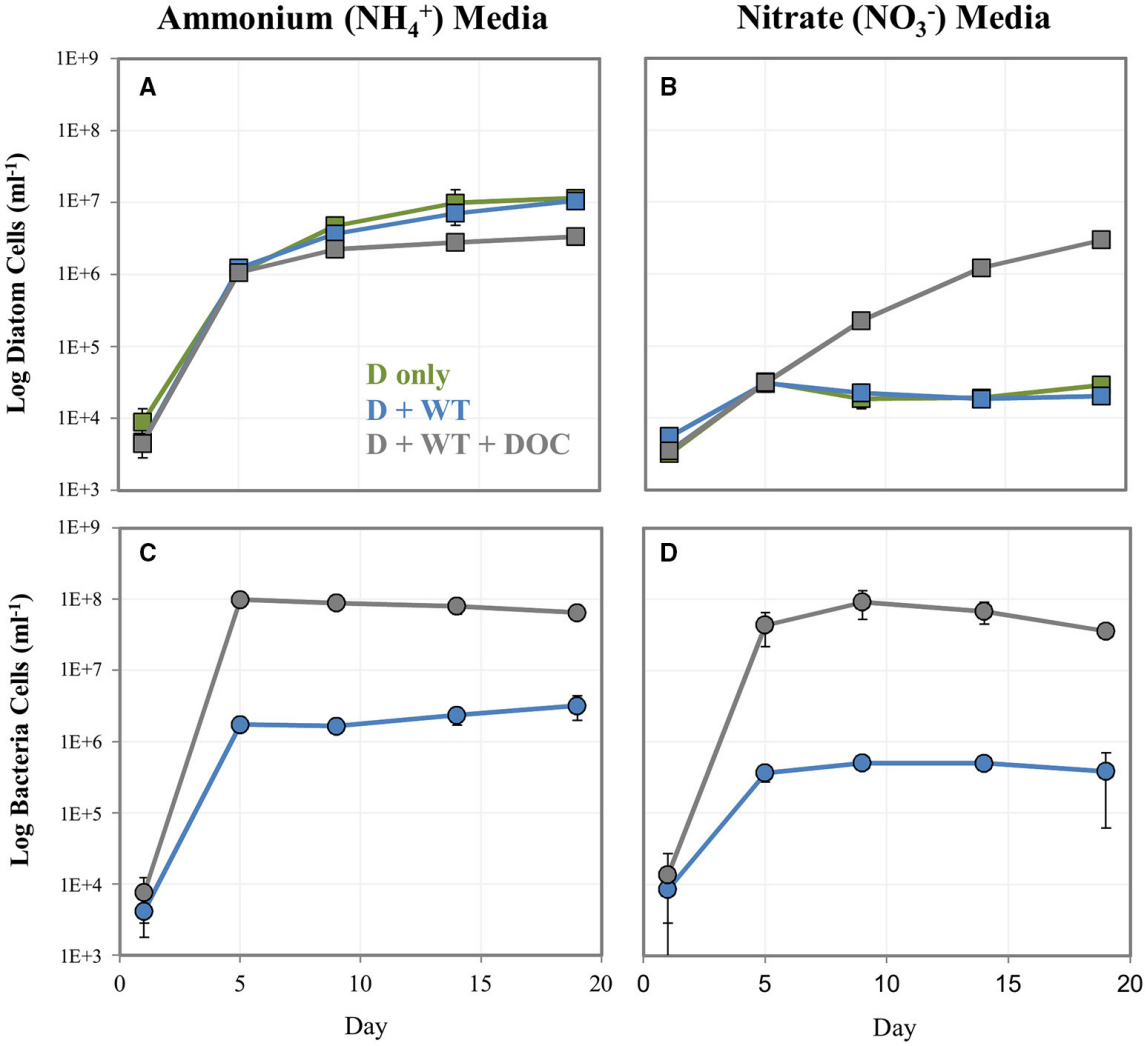
**Log cell numbers per ml of ***P. tricornutum*** NRKO (A,B) and ***A. macleodii*** WT bacteria (C,D) in co-culture, grown on ${\text{NH}}_{4}^{+}$ or ${\text{NO}}_{3}^{-}$ and as the sole nitrogen source**. Green markers = *P. tricornutum* NRKO monoculture (also noted as D only), blue = *P. tricornutum* NRKO + *A. macleodii* WT (also noted as D + WT), and gray = *P. tricornutum* NRKO + *A. macleodii* WT + DOC (also noted as D + WT + DOC).

We explored whether bacteria can provide diatoms with a useable N source and potentially “rescue” N-starved diatoms. To do this, we cultured the NRKO diatom strain (lacking the ability to use ${\text{NO}}_{3}^{-}$) in media containing either ${\text{NH}}_{4}^{+}$ or ${\text{NO}}_{3}^{-}$ as the sole N source, in the presence of absence WT *A. macleodii*, and with the bacteria and a DOC addition at T_o_ (Figure 4). The NRKO diatom was able to grow in all treatments with ${\text{NH}}_{4}^{+}$ as the provided N source, and in the absence of added DOC grew similarly with and without the addition of *A. macleodii* (Figures 4A,B). In ${\text{NH}}_{4}^{+}$ media amended with DOC, bacteria numbers were much higher, and NRKO diatom cell densities were lower (Figures 4C,D), an observation similar to the DOC addition experiment described above. With ${\text{NO}}_{3}^{-}$ as the sole N source and without DOC addition, the diatom NRKO strains displayed little detectable growth with and without *A. macleodii* addition. However, the NRKO diatom-WT bacteria co-culture to which DOC was added displayed a much different growth response; diatom density increased linearly (*R*^2^ = 0.96) throughout the experiment, with a maximum cell density of 3.0 × 10^6^ cells ml^−1^ measured on day 19 of the experiment (Figures 4A,B). Bacterial cell densities in this treatment peaked on day 9 of the experiment, and subsequently declined.

### Effect of various ${\text{NO}}_{3}^{-}$ and DOC concentrations on diatom-bacteria population dynamics

**Figure 5:**
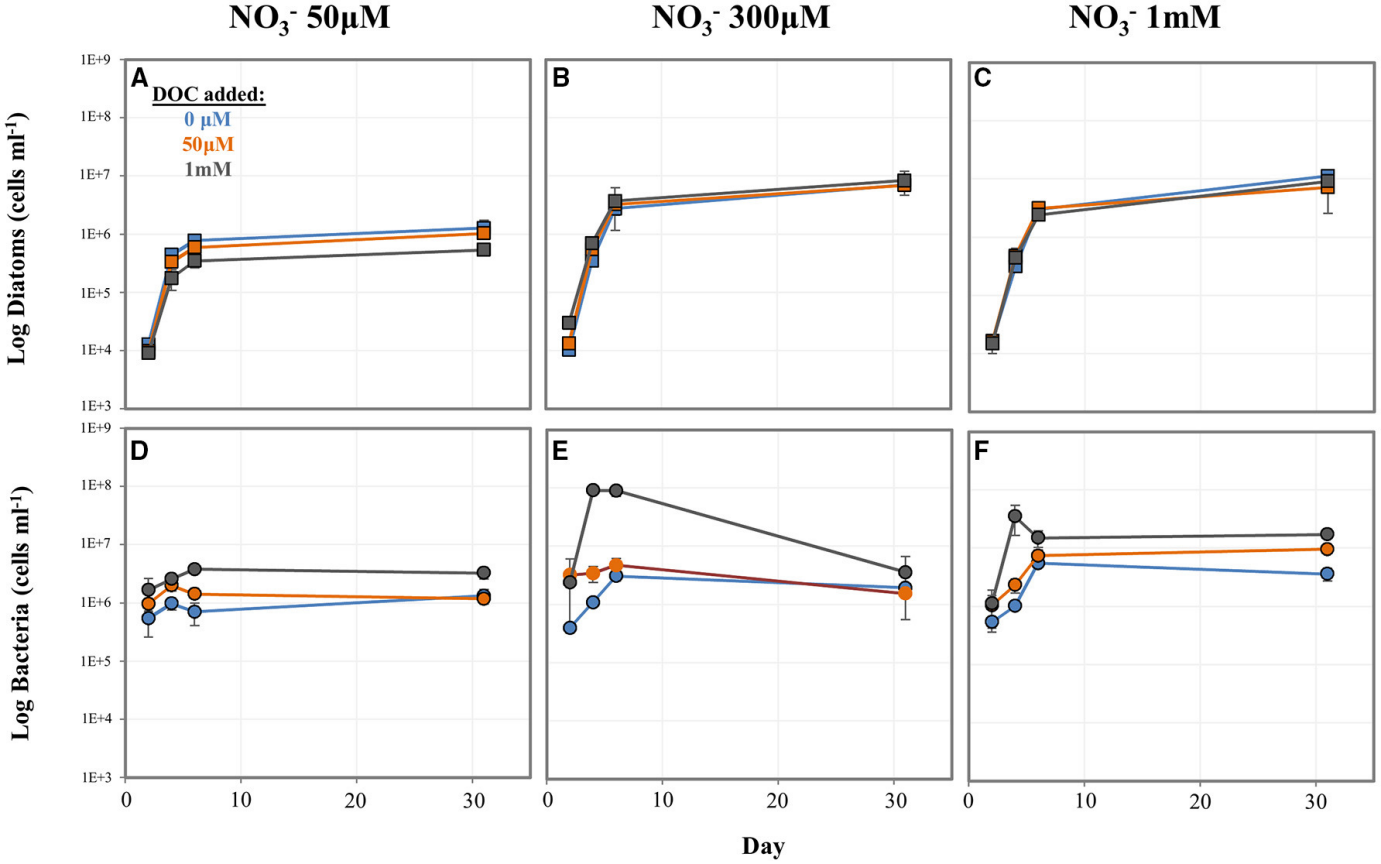
**Log cell numbers per ml of ***P. tricornutum*** and ***A. macleodii*** grown in co-culture under variable nitrate and dissolved organic carbon (DOC) concentrations**. Diatom cell numbers are shown in **(A–C)** and bacterial numbers shown in **(D–F)**. Blue = 0 μM DOC added, orange = 50 μM DOC added, and gray = 1 mM DOC added.

To better understand the influence of N and C concentration on diatom-bacteria growth dynamics, we conducted a factorial experiment in which diatom-WT bacteria co-cultures were grown in 9 different media types encompassing a range of ${\text{NO}}_{3}^{-}$ and DOC levels. In general, higher ${\text{NO}}_{3}^{-}$ levels resulted in higher numbers of both diatoms and bacteria. DOC concentrations had a strong effect on bacterial cell numbers, but less of an effect on diatom concentrations except for at low ${\text{NO}}_{3}^{-}$. At the lowest ${\text{NO}}_{3}^{-}$ concentration tested (50 μM), diatom cell numbers decreased with increasing DOC concentration (Figure 5A) while bacterial cell numbers increased (Figure 5D). However, both diatom and bacteria concentrations were low at low ${\text{NO}}_{3}^{-}$ level regardless of DOC concentration compared to the higher ${\text{NO}}_{3}^{-}$ treatments. At the higher ${\text{NO}}_{3}^{-}$ levels (300 μM and 1 mM), *P. tricornutum* concentrations were higher and similar to each other across DOC treatments (Figures 5B,C). In almost all treatments, *A. macleodii* concentrations were higher during the *P. tricornutum* exponential phase compared to the stationary sampling point on day 31 (Figures 5D–F). The reverse was true with the diatoms; cell numbers increased between day 6 and day 31 (Figures 5A–C). Final *A. macleodii* cell densities measured on day 31 were positively correlated with both DOC and ${\text{NO}}_{3}^{-}$ concentration: lower concentrations were observed at the lower levels of both ${\text{NO}}_{3}^{-}$ (50 and 300 μM) and DOC (0 and 50 μM), and highest concentrations were observed in the high ${\text{NO}}_{3}^{-}$, high DOC cultures and (Figures 5D–F).

In media containing 300 μM ${\text{NO}}_{3}^{-}$ and no DOC addition (the same conditions as the baseline experiment), *P. tricornutum* maintained in semi-continuous cultures displayed consistent growth rates across 5 rounds of culture transfer, indicating steady-state growth. The average μ = 1.42, with a range of μ = 1.25 to 1.62 (Supplementary Table 6).

## Discussion

### A continuum: Diatom-bacteria ${\text{NO}}_{3}^{-}$ utilization and the role of organic DOC availability

Since phytoplankton and bacteria often co-occur in the same environments, the concept of competition for nutrients has been of interest for decades (Bratbak and Thingstad, 1985; Thingstad et al., 1993; Logan et al., 1995; Grossart and Simon, 2007; Amin et al., 2012). New relationships between phytoplankton and bacteria are being discovered and investigated with increasing frequency (Durham et al., 2014; Amin et al., 2015; Bertrand et al., 2015; Smriga et al., 2016), in part due to recent methodological advances. In particular, *Alteromonas* bacteria have been shown to have positive, negative, and seemingly neutral relationships with individual phytoplankton species. It is unclear, however, what role competition for nutrients may play in these observed interactions Understanding the heterogeneous landscape in which these interactions occur and the resulting impacts on global biogeochemical cycles, particularly carbon and nitrogen cycling, is an active area of research (Stocker, 2012; Worden et al., 2015; Smriga et al., 2016).

Many phytoplankton-bacteria interactions likely fall somewhere along a commensal-competitive continuum. Phytoplankton release increasing amounts of organic matter when nutrients become limited, stimulating the growth of bacteria which may compete with them for the same limiting nutrients, an apparent paradox (Bratbak and Thingstad, 1985; Bertrand et al., 2015). Studies examining acquisition of inorganic phosphate have found that bacteria can compete with phytoplankton, and that this potential competition can be nutrient concentration dependent (Bratbak and Thingstad, 1985; Thingstad et al., 1993), and bacteria-phytoplankton population dynamics may be dependent on what species are present as well as nutrient concentrations (Puddu et al., 2003; Grossart and Simon, 2007). Interestingly, while inorganic N availability is also a major driver in ocean productivity and biogeochemical cycling, and both phytoplankton and many bacteria can utilize it, few studies have examined these potentially important interactions (Dugdale and Goering, 1967; Allen et al., 2001; Amin et al., 2012; Jiang et al., 2015). This is despite findings that bacterial *nasA* genes are common, diverse, and highly expressed during phytoplankton blooms, especially for particular bacterial classes and genera (including *Alteromonas*) (Allen et al., 2012; Jiang et al., 2015). Recent advancements in microbiology, including genetic manipulation and the introduction of powerful next generation sequencing technology, call for an examination of these relationships with an aim to understand the underlying complex cellular mechanisms.

Utilizing both WT and Δ*nasA* bacteria in co-culture with diatoms presents the opportunity to explore how the bacteria and diatom in this model system impact one another and are impacted by ${\text{NO}}_{3}^{-}$ availability and utilization. ${\text{NO}}_{3}^{-}$ was drawn down quickly in the experiments, and the diatom population continued to increase in all co-cultures even after the complete depletion of ${\text{NO}}_{3}^{-}$ from the media, which is consistent with reports of the ability of diatoms to rapidly accumulate and store N present in the environment (Cermeño et al., 2011). During exponential phase with no DOC added to the system, *P. tricornutum* growth rate, cell number, and other aspects of *P. tricornutum* physiology, including growth rate, Chl a concentration, Fv/Fm, and culture pH were not affected by the presence of either bacterial strain (Figure 1, Supplementary Figure 2, Supplementary Tables 1, 3). At this sampling point, bacterial numbers began to increase after an initial decrease in the experiment. One possible explanation for these observations is that during the exponential phase, the diatom and bacteria co-exist in a commensal relationship whereby the diatom is not affected by the presence of bacteria, but the bacteria are able to benefit from diatom organic matter. However, it is also possible that low bacterial cell numbers and biomass obscure any positive or negative effect that the bacteria may be having on the diatoms on a smaller scale, and as a result differences are not observable based on the methods we used in this study. Later in the diatom's stationary phase, when nitrogen is limited, cultures containing bacteria reached higher cell densities than the *P. tricornutum* monocultures while bacterial numbers also continued to increase, suggesting a potential cooperative relationship. We hypothesize that the increase in diatom cell number is the result of bacteria providing diatoms with a viable nitrogen source in exchange for DOC (discussed further below).

Previous studies have examined growth effects of *Alteromonas* bacteria in co-culture with phytoplankton. *Alteromonas* bacteria species cultured with eukaryotic phytoplankton have been shown to exhibit algicidal (thought to be the result of secreted dissolved substances) and non-algicidal effects (reviewed in Mayali and Azam, 2004). When cultured with the prokaryotic cyanobacteria from the *Prochlorococcus* genus, the presence of *Alteromonas* sometimes provide the algae with benefits by protecting them from oxidative stress (Morris et al., 2008, 2011) while in other cases they can cause growth inhibition (Sher et al., 2011; Aharonovich and Sher, 2016). In prior non-*Alteromonas* co-culturing studies, dynamics between phytoplankton and bacteria have been shown to manifest late in the growth cycle (Grossart and Simon, 2007; Wang et al., 2014), though often the result is commensal bacteria turning algicidal, possibly to relieve nutrient stress. Immediate growth increases in diatom populations before and during exponential phase resulting from the presence of bacteria have also been observed (Grossart and Simon, 2007; Amin et al., 2015). These results are for the most part different from what we observed in our study, which may be due to the differences in the species of both phytoplankton and bacteria examined (even within the genus *Alteromonas*, species are quite diverse and were not always identified in these studies). Despite being observed in a prokaryote-prokaryote co-culturing system, the possibility that *Alteromonas* bacteria may protect phytoplankton from oxidative stress is interesting and could be examined in the future using the model system developed in this study.

While the presence of the bacteria alone did not hinder diatom growth, when DOC was added early in the diatom growth phase (prior to the depletion of ${\text{NO}}_{3}^{-}$ from the media) the bacteria could acquire ${\text{NO}}_{3}^{-}$ from the media, making it unavailable to the diatoms. Our data suggest that depending on the availability of allochthonous DOC, bacteria that have the ability to utilize ${\text{NO}}_{3}^{-}$ in the environment are able to effectively compete for ${\text{NO}}_{3}^{-}$. This pattern was not observed in co-cultures containing Δ*nasA* bacteria, illustrating how bacteria that are unable to use ${\text{NO}}_{3}^{-}$ cannot take advantage of surplus organic C in ${\text{NO}}_{3}^{-}$ cultures, and in the case of Δ*nasA* appear to be limited by diatom C production. The WT bacteria generally maintained higher cell numbers than the Δ*nasA* bacteria, possibly suggesting that the WT bacteria were limited by C while the Δ*nasA* bacteria were limited by N. However, the addition of DOC to Δ*nasA* bacteria co-cultures did result in a slight bacterial growth increase (Figure 3B). Thus, it is possible that a co-limitation scenario may also arise in low concentrations of both bioavailable N and C.

When diatoms interact with bacteria that can utilize ${\text{NO}}_{3}^{-}$, the concentration of both ${\text{NO}}_{3}^{-}$ and DOC present may impact their dynamics in complex ways. To explore this, we tested the effects of various ${\text{NO}}_{3}^{-}$ and DOC levels on population dynamics of *P. tricornutum*–*A. macleodii* co-cultures. We observed that *P. tricornutum* cell densities were largely regulated by ${\text{NO}}_{3}^{-}$ concentrations rather than DOC level or bacteria cell densities. An exception was at a low ${\text{NO}}_{3}^{-}$ concentrations (50 μM), where bacterial accumulation of ${\text{NO}}_{3}^{-}$ linked to increased bacteria cell numbers appeared to reduce diatom growth and final cell densities. This trend was not observed in the higher ${\text{NO}}_{3}^{-}$ treatments, where diatom cell densities were similar between 50 μM and 1 mM DOC concentrations despite an increase in bacteria cell density. This suggests that at low ${\text{NO}}_{3}^{-}$ levels, bacterial growth made possible by DOC availability can have a negative effect on diatom cell numbers. Based on a similar pattern observed in the DOC addition study discussed above, where ${\text{NO}}_{3}^{-}$ drawdown was correlated with high bacterial and low diatom cell numbers in high DOC conditions, we hypothesize that in this experiment fast bacterial acquisition of ${\text{NO}}_{3}^{-}$ in the media made ${\text{NO}}_{3}^{-}$ limiting for diatom growth. At higher ${\text{NO}}_{3}^{-}$ concentrations this effect was not observed, which could be explained by the diatoms having sufficient opportunity to acquire and store N since more was available. The diatoms may also be able to utilize nitrogen derived from dead or growth-arrested bacteria during stationary phase. This is supported by the observation that bacterial abundance peaked during the exponential phase of *P. tricornutum* regardless of ${\text{NO}}_{3}^{-}$ level or DOC addition and subsequently declined, with the exception of the 1 mM ${\text{NO}}_{3}^{-}$/50 μM DOC treatment.

Bacterial cell numbers were strongly affected by DOC concentration. Without the addition of DOC, bacterial cell densities generally increased with increasing ${\text{NO}}_{3}^{-}$ and corresponding increases in diatom cell density. This likely reflects the link between diatom population and DOC availability; higher diatom density as a result of higher N concentrations leads to higher total DOC available for bacteria utilization. It is also possible that changes in DOC composition may affect bacterial growth dynamics. At higher DOC concentrations, bacteria could utilize ${\text{NO}}_{3}^{-}$ in the media to reach higher cell densities than with ambient DOC alone, a result consistent with other DOC addition experiments conducted in this study. By late stationary phase (Day 31), bacteria cell densities were variable. This may be the result of complex N and C recycling dynamics following initial uptake by bacteria and diatoms, and further studies may help to elucidate how cellular responses of diatoms and bacteria lead to the population changes we observed.

### Exchange of nitrogen substrates between diatoms and bacteria

Our findings suggest that the diatoms and bacteria in our model system are able to exchange nitrogen substrates with each other. A large portion of N consumed by phytoplankton is ultimately released as dissolved organic nitrogen (DON) in oceanic, coastal, and estuarine environments (Wheeler and Kirchman, 1986; Bronk et al., 1994; Berman and Bronk, 2003), and is a valuable source of N for marine bacteria. Previous studies on *P. tricornutum* have shown that both organic C and N are released by *P. tricornutum*, and that concentrations increase after the cells enter stationary phase (Chen and Wangersky, 1996; Pujo-Pay et al., 1997). The bacteria in the present study were able to survive and grow in co-culture with the diatom using diatom-derived organic C, and bacteria concentrations increased after the onset of diatom stationary phase, which is consistent with utilization of diatom derived organic matter. We further observed that the Δ*nasA* bacteria strain in co-culture grew despite the inability to use ${\text{NO}}_{3}^{-}$, which was the only N source provided in the ASW media. This leaves diatom-derived N as the most plausible source for bacterial growth.

While the paradigm for phytoplankton-bacteria relationships is typically that of bacteria utilizing phytoplankton-derived organic matter, and in some cases exchanging various substrates to facilitate this acquisition, few studies have examined if and how diatoms may use bacterial-derived N. Given the high rate of bacterial turnover in the ocean, this could potentially represent an important N source, especially under N limiting conditions. Diatoms are known to utilize a variety of organic and inorganic N substrates (Bronk et al., 1994, 2006; Waser et al., 1998). One recent study suggests that bacteria may use ${\text{NH}}_{4}^{+}$ as a diatom signaling molecule (Amin et al., 2015). Our results show that the NRKO diatom lines could survive and grow normally in ${\text{NH}}_{4}^{+}$ but not ${\text{NO}}_{3}^{-}$. The addition of *A. macleodii* to the diatom cultures without a coincident DOC media amendment resulted in low bacterial cell numbers and did not increase NRKO diatom cell numbers, suggesting that either the bacteria do not provide the diatom with useable N substrates in this physiological state, or that the amount supplied is not sufficient for diatom growth. Potentially, the bacteria could provide the diatoms with useable N, but the amount produced by the low cell numbers observed was not enough to detect diatom population recovery using our methods. Our study does not address other possible interactions at the cellular level (e.g., metabolic shifts perhaps observable using metabolomics or transcriptomics), which may clarify whether N is being provided by bacteria in this co-culture. When DOC was added to the NRKO co-cultures in ${\text{NO}}_{3}^{-}$ media, both bacteria and diatom growth increased substantially. This strongly suggests that the bacteria supply the diatoms with a N substrate, perhaps made possible by the large bacterial numbers resulting from the DOC addition, followed by nitrogen release upon the onset of carbon-limited stationary phase. Alternatively or concurrently, after reaching maximum cell density early in the experiment the bacteria may subsequently die allowing *P. tricornutum* to recycle some of the organic N from the dead bacterial cells. Some bacteria were detected in the *P. tricornutum* NRKO cultures without the addition of *A. macleodii*. The addition of DOC to *A. macleodii* amended cultures resulted in high bacterial densities similar to what was observed in the *P. tricornutum* WT–*A. macleodii* co-cultures (Figure 3), thus we believe *A. macleodii* growth was responsible for the observed increase in *P. tricornutum* NRKO cell numbers. Even if this is not the case (i.e., the growth of other bacteria present contributed to the high bacterial cell numbers), our results strongly suggest that the high abundance of bacteria due to DOC addition was linked to diatom recovery, and our study presents a proof of concept that diatoms can utilize bacterial-derived N. Though the scope of this study does not directly address the specific bacterial N source, the transcriptomic analysis conducted during the baseline experiment elucidates N-related diatom cellular pathways that may be influenced by bacteria, such as those involved in ${\text{NO}}_{3}^{-}$ and ${\text{NH}}_{4}^{+}$ acquisition and metabolism.

### Further insights into “bacterial responsive” genes revealed by RNA-seq

Using RNA-seq, we were able to identify several putative *P. tricornutum* genes that were responsive to the presence of bacteria, some of which suggest N-related interactions. A recent study examined the response of two *Prochlorococcus* strains in co-culture with *A. macleodii* bacteria, and they also found that several algal genes were bacterial-responsive (Aharonovich and Sher, 2016). The study examines prokaryotic rather than eukaryotic gene expression, and is thus difficult to compare. However, it demonstrates the value of transcriptome analyses in developing hypotheses about microbial interactions and, along with our study, lays the framework for a robust analysis of interactions involving *A. macleodii* bacteria with both prokaryotic and eukaryotic algae in a model laboratory system.

All differential gene expression in our study was observed in the stationary samples, long after ${\text{NO}}_{3}^{-}$ had been depleted from the media (Figure 1), and cells were potentially N stressed. One discernable pattern is related to downregulation of multiple N transporters (${\text{NO}}_{2}^{-}$ and ${\text{NO}}_{3}^{-}$) in diatom cultures containing bacteria (Figure 2, Supplementary Table 4). One of these transporters (Phatr2ID: 49093, Phatr3 ID: 302957) was one of the mostly highly significantly DE putative genes identified in the data set. Orthologs of this ion transporter in *Arabidopsis* has been shown to bind to and sense ${\text{NO}}_{3}^{-}$ (Huang et al., 1996; Liu et al., 1999), suggesting a possible role in diatom ${\text{NO}}_{3}^{-}$ acquisition. Inorganic N transporters including those DE in our dataset are commonly upregulated during nitrogen stress conditions (Levitan et al., 2014; Alipanah et al., 2015; JGI genome annotation, Allen, 2006). Downregulation in cultures containing bacteria suggests that bacteria are contributing to alleviation of diatom N stress, which is further supported by the higher diatom cell numbers observed in co-cultures during stationary phase and also our finding that under certain N stress conditions (i.e., high DOC present) bacteria have the ability to provide diatoms with N in forms other than ${\text{NO}}_{3}^{-}$. In Levitan et al. (2014), expression of glutamine synthetase II (GSII) followed a similar expression pattern of downregulation under N-stress, and we also observed significant downregulation of this gene in bacteria-containing co-cultures. Another gene related to diatom N metabolism, ornithine cyclodeaminase (Phatr2: 54222, Phatr3: 305662), is involved in the diatom Urea cycle and was downregulated compared to *P. tricornutum* monocultures (Allen et al., 2011). While these two genes were only significantly DE between the *P. tricornutum* monoculture and the Δ*nasA* bacterial co-cultures (Supplementary Table 4), they were downregulated in both co-cultures indicating a common bacterial-responsive pattern.

Some ${\text{NH}}_{4}^{+}$ transporters have also been shown to be upregulated during N stress, while others are downregulated or unaffected (Levitan et al., 2014; Alipanah et al., 2015). Examination of ${\text{NH}}_{4}^{+}$ acquisition and transport genes in our dataset did not reveal any significant DE between *P. tricornutum* monocultures and co-cultures (Supplementary Table 5). In Amin et al. (2015), it was suggested that bacteria provide ${\text{NH}}_{4}^{+}$ to diatoms as a signaling molecule, which was partially supported by the upregulation of ${\text{NH}}_{4}^{+}$ transport genes in bacteria, as well as an increase in ${\text{NH}}_{4}^{+}$ measured in co-cultures. However, ${\text{NH}}_{4}^{+}$ transport genes were not DE in the diatom they examined, which may suggest that the cellular impacts of this potential exchange are not apparent at the transcriptional level in diatoms.

Several other bacterial responsive genes, including the many unannotated hypothetical proteins in our dataset, may be interesting candidates for further investigation. Several heat shock transcription factors were upregulated in diatom-bacteria co-cultures compared to monocultures. These are transcriptional regulators of heat shock proteins involved in cellular stress responses (Sorger, 1991). High expression may indicate a stress response caused by the bacteria, however, little is known about the regulation of this complex pathway in diatoms, and many non-transcriptional steps are involved in heat shock protein expression and regulation (Sorger, 1991). Upregulation of other putative genes in co-cultures may play a role in exchange of important metabolites or intracellular signaling pathways. These include a putative sugar transporter (Phatr2 ID: 49722, Phatr3 ID: 311238) which was >2 fold upregulated in *P. tricornutum* in both co-cultures (Supplementary Table 4), and the two putative genes that were most highly upregulated and significantly DE in both co-cultures: a putative short chain dehydrogenase (Phatr2 ID: 13001, Phatr3 ID: 306282) and a putative fatty acid hydroxylase (Phatr3 ID: 308140). Short chain dehydrogenases in particular have been shown to serve as molecular links between nutrient signaling and plant hormone biosynthesis in *Arabidopsis* (Cheng, 2002). Further analysis using our model system may allow determination of the role of such genes in diatom-bacteria interactions.

Using the genetically tractable model system developed in this study, we have described mechanisms of interaction between diatoms and bacteria that may be of global biogeochemical significance. Our data strongly suggests bidirectional exchange of N substrates between diatoms and bacteria, and revealed putative diatom genes and pathways that may be impacted by the presence of bacteria and involved in N exchange. Furthermore, we have demonstrated that under certain environmental conditions (i.e., high DOC), marine bacteria are able to effectively compete with diatoms for ${\text{NO}}_{3}^{-}$, which may influence predictions of primary productivity and nutrient utilization by phytoplankton in the ocean and associated estimates of C export via the biological pump.

## Author contributions

RD and AA designed research; RD, SS, and HZ performed research; RD, SS, and JM analyzed data; and RD and AA wrote the paper.

## Funding

Funding was provided to AA by the Gordon and Betty Moore Foundation (GBMF3828 and GBMF5006), the US Department of Energy (DE-SC0008593) and the National Science Foundation (OCE-1136477). Funding was provided to RD by the UCSD/SIO Center for Marine Biodiversity and Conservation.

### Conflict of interest statement

The authors declare that the research was conducted in the absence of any commercial or financial relationships that could be construed as a potential conflict of interest.
