# Supplementary material for: Genetic Manipulation of Competition for Nitrate between Heterotrophic Bacteria and Diatoms
Source: Front Microbiol. 2016 Jun 9;7:880. doi: 10.3389/fmicb.2016.00880 (PMC4899447; doi:10.3389/fmicb.2016.00880)
Supplement: Supplementary file 1 [file Table1.PDF]

Supplementary Table 1: Specific growth rate (μ) of *P. tricornutum* in monoculture, in co-culture with WT *A. macleodii* bacteria, and in co-culture with Δ*nasA A. macleodii* bacteria. Values are presented the average of n = 3 biological replicates ± the standard deviation.

| **Treatment** | **Specific Growth Rate (μ)** |
|---|---|
| ***P. tricornutum*** **monoculture** | 1.37 ± 0.10 |
| ***P. tricornutum-A. macleodii*** **WT co-culture** | 1.34 ± 0.04 |
| ***P. tricornutum-A. macleodii*** **Δ*nasA*** **co-culture** | 1.28 ± 0.06 |
