# Supplementary material for: Genetic Manipulation of Competition for Nitrate between Heterotrophic Bacteria and Diatoms
Source: Front Microbiol. 2016 Jun 9;7:880. doi: 10.3389/fmicb.2016.00880 (PMC4899447; doi:10.3389/fmicb.2016.00880)
Supplement: Supplementary file 2 [file Table2.PDF]

Supplementary Table 2. Cell numbers and media nitrate concentrations from the baseline experiment (also shown in Figure 1). Average treatment diatom and bacteria cells per ml measured by flow cytometry, and nitrate concentrations in µM measured by UV spectrophotometry for each day of the experiment. N/A represents data not collected because it was not applicable. n.d. indicates samples that were measured but concentrations were not detectable. N=3 replicate cultures.

Experiment Day	Treatment	Day	Average Diatom Cells ml $^{-1}$	St. Dev.	Average Bacteria Cells ml $^{-1}$	St. Dev	Average NO3 Concentration (µM)	St. Dev.
1	Diatom Monoculture	1	7049	1679	N/A	N/A	244.46	2.11
1	Diatom + WT bacteria	1	14063	2989	52430	9115	246.48	2.43
1	Diatom + Δ*nasA* bacteria	1	9489	2710	24962	4982	244.28	4.24
2	Diatom Monoculture	2	28237	2778	N/A	N/A	240.12	2.58
2	Diatom + WT bacteria	2	36048	803	388188	82182	238.80	2.03
2	Diatom + Δ*nasA* bacteria	2	35212	919	100702	75939	243.23	5.08
3	Diatom Monoculture	3	89712	9559	N/A	N/A	221.51	0.73
3	Diatom + WT bacteria	3	102523	58005	526333	284914	220.54	2.26
3	Diatom + Δ*nasA* bacteria	3	88324	17501	129016	93726	224.55	1.90
4	Diatom Monoculture	4	308560	53817	N/A	N/A	162.12	7.10
4	Diatom + WT bacteria	4	330332	11696	257669	68586	163.11	4.58
4	Diatom + Δ*nasA* bacteria	4	374136	79849	128568	71996	166.01	6.22
5	Diatom Monoculture	5	1753140	353600	N/A	N/A	4.72	2.65
5	Diatom + WT bacteria	5	1998932	238317	989477	330780	n.d.	n.d.
5	Diatom + Δ*nasA* bacteria	5	1638305	328453	446099	409032	3.07	2.69
6	Diatom Monoculture	6	2571921	208872	N/A	N/A	n.d.	n.d.
6	Diatom + WT bacteria	6	2862348	201484	703792	206355	n.d.	n.d.
6	Diatom + Δ*nasA* bacteria	6	3133224	249496	276554	231566	n.d.	n.d.
8	Diatom Monoculture	8	3620453	706867	N/A	N/A	n.d.	n.d.
8	Diatom + WT bacteria	8	4354149	559715	880163	190250	n.d.	n.d.
8	Diatom + Δ*nasA* bacteria	8	5258613	997342	342028	158426	n.d.	n.d.
10	Diatom Monoculture	10	6049204	1252589	N/A	N/A	n.d.	n.d.
10	Diatom + WT bacteria	10	7714340	161896	1009980	13197	n.d.	n.d.
10	Diatom + Δ*nasA* bacteria	10	9080393	337354	665910	161685	n.d.	n.d.
11	Diatom Monoculture	11	6258265	880125	N/A	N/A	n.d.	n.d.
11	Diatom + WT bacteria	11	5752566	976161	1192969	238398	n.d.	n.d.
11	Diatom + Δ*nasA* bacteria	11	5059108	1109161	720786	347512	n.d.	n.d.

13	Diatom Monoculture	13	5068824	98254	N/A	N/A	n.d.	n.d.
13	Diatom + WT bacteria	13	5663779	315454	2616209	191161	n.d.	n.d.
13	Diatom + Δ*nasA* bacteria	13	5715158	646403	1903525	805749	n.d.	n.d.
15	Diatom Monoculture	15	5752518	533370	N/A	N/A	n.d.	n.d.
15	Diatom + WT bacteria	15	7217243	958403	2099876	247228	n.d.	n.d.
15	Diatom + Δ*nasA* bacteria	15	6194411	687923	1676805	394639	n.d.	n.d.
19	Diatom Monoculture	19	5607077	829614	N/A	N/A	n.d.	n.d.
19	Diatom + WT bacteria	19	8246963	1003139	4442598	225679	n.d.	n.d.
19	Diatom + Δ*nasA* bacteria	19	8959981	527982	3919447	892425	n.d.	n.d.
