# Supplementary material for: Genetic Manipulation of Competition for Nitrate between Heterotrophic Bacteria and Diatoms
Source: Front Microbiol. 2016 Jun 9;7:880. doi: 10.3389/fmicb.2016.00880 (PMC4899447; doi:10.3389/fmicb.2016.00880)
Supplement: Supplementary file 3 [file Table3.PDF]

Supplementary Table 3: Physiological data collected during the exponential and stationary time points for *P. tricornutum* in monoculture, in co-culture with WT *A. macleodii* bacteria, and in co-culture with Δ*nasA A. macleodii* bacteria. Measurements were taken for Chlorophyll A (Chl-a), Fv/Fm, pH, dissolved organic carbon (DOC), particulate organic carbon (POC), dissolved organic nitrogen (DON), and particulate organic nitrogen (DON). Values are presented the average of n = 3 biological replicates ± the standard deviation.

	**Chl-A (ug/L)**	**Fv/Fm**	**pH**	**DOC (μM)**	**POC (μM)**	**DON (μM)**	**PON (μM)**
**Exponential Sampling Point (Day 5)**							
***P. tricornutum* Only**	277 ± 38	0.62 ± 0.02	9.99 ± 0.08	1.49 ± 0.14	1.34 ± 0.36	*0.25 ± 0.02*	0.17 ± 0.06
***P. tricornutum* + *A. macleodii* WT**	248 ± 24	0.65 ± 0.01	9.94 ± 0.08	1.46 ± 0.16	1.67 ± 0.18	*0.24 ± 0.02*	0.21 ± 0.04
P. tricornutum* + *A. macleodii* Δ*nasA	233 ± 25	0.63 ± 0.02	9.98 ± 0.10	1.42 ± 0.11	1.44 ± 0.40	*0.25 ± 0.01*	0.21 ± 0.02
**Stationary Sampling Point (Day 13)**							
***P. tricornutum* Only**	145 ± 18	0.33 ± 0.02	9.48 ± 0.09	1.42 ± 0.01	4.07 ± 0.32	*0.35 ± 0.00*	0.29 ± 0.03
***P. tricornutum* + *A. macleodii* WT**	133 ± 28	0.32 ± 0.02	9.53 ± 0.05	1.34 ± 0.10	4.19 ± 0.34	*0.33 ± 0.02*	0.31 ± 0.02
P. tricornutum* + *A. macleodii* Δ*nasA	152 ± 6	0.30 ± 0.03	9.46 ± 0.04	1.36 ± 0.07	4.07 ± 0.29	*0.33 ± 0.01*	0.31 ± 0.04
