# Supplementary material for: Genetic Manipulation of Competition for Nitrate between Heterotrophic Bacteria and Diatoms
Source: Front Microbiol. 2016 Jun 9;7:880. doi: 10.3389/fmicb.2016.00880 (PMC4899447; doi:10.3389/fmicb.2016.00880)
Supplement: Supplementary file 4 [file Table4.PDF]

Supplementary Table 4: Putative *P. tricornutum* genes that were significantly differentially expressed between *P. tricornutum* monocolutures and only one of the WT or Δ*nasA* bacterial co-cultures, and fold expression change for the significantly different comparison. Average reads per kilobase of transcript per million mapped reads (RPKM) for each treatment (*P. tricornutum* monoculture, *P. tricornutum-A. macleodii* WT co-culture, and *P. tricornutum-A. macleodii* Δ*nasA* co-culture) are also shown to allow identification of "bacterial specific" trends. Gene IDs are shown for both Phatr2 and Phatr3.

**Phatr3 Gene ID**	**Phatr 2 Gene ID**	**Gene Annotation**	**Fold Change**	***P. tricornutum* (RPKM Ave.)**	***P. tricornutum +A. macleodii* WT (RPKM Ave)**	***P. tricornutum +A. macleodii* Δ*nasA* (RPKM Ave)**	**Additional Gene Information**
**Significantly DE between *P. tricornutum* monocultures and *P. tricornutum-A. macleodii* WT co-cultures**							
308281	13076	Nitrite Transporter	2.99	64.68	20.22	33.64	Homology to *Chlamydomonas reinhardtii* nitrate transporter NAR1; strongly expressed under low CO2 conditions.
307720	26029	Nitrate Transporter	2.89	220.45	70.24	108.08	Membrane associated high-affinity nitrate transporter responsible for the active transport of nitrate into the cell. High EST support for expression under nitrate depletion conditions.
311709	44835	Predicted E3 ubiquitin ligase	1.76	100.54	41.71	60.73	Regulates diverse cellular functions including cell trafficking, DNA repair, and signalling.
310165	46177	Hypothetical protein	1.64	238.25	102.31	99.81	
305781	44838	Predicted E3 ubiquitin ligase	1.59	120.22	52.15	78.76	Regulates diverse cellular functions including cell trafficking, DNA repair, and signalling.
300826	8987	Mitochondrial phosphate carrier protein	-1.21	6.48	14.79	12.15	Catalyzes the transport of phosphate into the mitochondrial matrix

307276	16375	Pyrroline-5-carboxylate reductase	-1.30	36.55	85.48	43.79	Involved in the proline biosynthesis pathway
302274	47234	Hypothetical protein	-1.55	4.10	10.25	5.03	
307340	N/A	Hypothetical protein containing HMG box domain	-2.14	13.88	30.68	41.10	Involved in regulation of DNA-dependent processes such as transcription, replication, and DNA repair
**Significantly DE between *P. tricornutum* monocultures and *P. tricornutum-A. macleodii* Δ*nasA* co-cultures**							
308539	N/A	Hypothetical protein	3.70	1769.35	2346.13	470.24	
302479	N/A	Hypothetical protein	3.43	16.97	18.19	4.73	
307291	49871	Hypothetical protein	3.16	28.06	22.50	8.27	
307423	50408	Mitochondrial carnitine-acylcarnitine carrier protein	2.65	17.13	8.96	5.46	Mediates the transport of acylcarnitines of different length across the mitochondrial inner membrane from the cytosol to the mitochondrial matrix for their oxidation by the mitochondrial fatty acid-oxidation pathway.
303961	35862	Uncharacterized protein conserved in bacteria (DUF2256)	2.58	14.79	9.31	4.77	
310497	N/A	FOG: Zn-finger	2.57	40.32	48.90	13.03	
306624	51092	Glutamine synthetase	2.48	51.43	25.83	17.20	Enzyme playing an essential role in nitrogen metabolism by catalyzing the condensation of glutamate and ammonia to form glutamine.
300360	N/A	Hypothetical protein	2.25	8.67	10.85	3.00	
307004	43232	Hypothetical protein of bacterial origin	2.22	18.90	13.94	6.86	
309057	9606	Alpha-hemolysin	2.20	9.60	10.81	3.38	In humans, lipids and proteins that cause the lysis of red blood cells.

310088	45852	Tryptophan/tyrosine permease family	2.15	59.85	41.37	22.23	Integral membrane proteins involves in amino acid transport into the cell.
300325	N/A	Hypothetical protein	1.99	886.23	1120.61	331.76	
308162	27851	40s ribosomal protein s10	1.85	134.59	160.78	52.71	
308634	21970	Membrane biopteran-transport related protein	1.84	15.21	12.21	5.97	
303949	12411	FKBP-type peptidyl-prolyl cis-trans isomerase	1.78	11.20	12.23	4.39	Chapperone protein that prevents aggregation of unfolded or partially folded proteins, promoting correct folding
308123	N/A	Hypothetical protein	1.74	43.39	41.45	17.62	
305949	12088	FKBP-type peptidyl-prolyl cis-trans isomerase	1.70	14.24	13.12	5.74	Chapperone protein that prevents aggregation of unfolded or partially folded proteins, promoting correct folding
304763	15371	Bacterial membrane flanked domain	1.63	49.96	49.28	20.84	
303334	47774	Hypothetical protein	1.62	11.48	9.44	4.70	
301810	50886	40S ribosomal protein S14	1.56	179.66	217.88	76.22	
306480	37403	Hypothetical protein	1.52	509.85	583.88	214.67	
307039	48938	Hypothetical protein	1.51	34.63	35.38	14.81	
305662	54222	Ornithine cyclodeaminase/mu-crystallin family of bacterial origin	1.50	18.45	16.32	7.74	Catalyzes the deamination of ornithine to proline
306678	38534	Alkyl hydroperoxide reductase/peroxiredo	1.44	26.83	21.37	11.78	Antioxidant enzymes that mediate signal transduction in

		xin					mammalian cells.
308715	14785	Alkyl hydroperoxide reductase, thiol specific antioxidant and related enzymes	1.43	11.89	8.08	5.20	Antioxidant enzymes that mediate signal transduction in mammalian cells.
309922	34681	NAD dependent epimerase/dehydratase family	1.39	37.18	31.86	16.54	Domain found in proteins that utilise NAD as a cofactor and use nucleotide-sugar substrates for a variety of chemical reactions
308068	45919	Hypothetical protein	1.37	19.03	19.66	8.40	
301225	5271	Ubiquitin-conjugating enzyme	1.34	23.99	16.57	10.81	Catalyze attachment of ubiquitin to substrate proteins
300332	N/A	Predicted GTPase-activating protein	1.34	44.07	27.77	19.26	
300917	49091	Zinc carboxypeptidase	1.30	12.35	11.98	5.57	
302401	37569	Predicted methyltransferase	1.29	12.07	9.83	5.39	
301310	5720	Predicted nucleic acid-binding protein ASMTL	1.28	20.54	22.93	9.30	
301996	46143	Hypothetical protein	1.28	50.29	42.58	23.43	
308280	12985	Predicted peptidyl-tRNA hydrolase	1.26	30.15	25.90	13.90	involved in protein biosynthesis
304710	22459	Delta 6-fatty acid desaturase/delta-8 sphingolipid desaturase, Cytochrome b5-like Heme/Steroid binding domain	1.24	13.58	11.45	6.24	Involved in fatty acid biosynthesis
309772	N/A	40S ribosomal protein S3	1.22	55.50	70.47	25.76	
303939	45918	Dehydrogenases with different specificities (related to short-	1.21	11.77	11.10	5.50	

		chain alcohol dehydrogenases)					
309645	51609	Tyrosine aminotransferase	1.18	26.85	21.56	12.81	catalyzes the conversion of tyrosineto 4-hydroxyphenylpyruvate
306182	521_bd	Aminomethyl transferase	1.17	37.29	34.40	17.55	
309833	44747	Predicted E3 ubiquitin ligase	1.15	14.37	13.10	6.85	Regulates diverse cellular functions including cell trafficking, DNA repair, and signalling.
305514	33499	Hypothetical protein	1.15	46.13	42.88	21.97	
307051	48983	3-phosphoglycerate kinase	1.13	20.45	18.95	9.82	Enzyme involved in glycolyis
305277	50128	Hypothetical protein	1.12	749.81	419.29	346.07	
306685	48483	Hypothetical protein	1.12	16.91	14.34	8.08	
310309	46348	Hypothetical protein	1.12	35.26	24.70	17.03	
311548	44212	Hypothetical protein	1.11	13.06	11.55	6.28	
302348	47143	Hypothetical protein	1.10	18.60	13.95	9.02	
311420	9709	Acyl carrier protein/NADH-ubiquinone oxidoreductase, NDUFAB1/SDAP subunit	1.08	84.19	74.77	41.01	Mitochondrial espiratory chain enzyme involved in cellular respiration and oxidative phosphorylation.
petD	N/A	Hypothetical protein	1.07	1918.90	1706.20	915.38	
310824	N/A	Molecular chaperone (DnaJ superfamily)	1.06	16.80	20.21	8.22	Stimulates the ATPase activity of DnaK and associates with unfolded polypeptide chains to prevent aggregation
306826	48859	Hypothetical protein	1.04	35.95	32.66	17.85	
302897	24996	Vesicle coat complex COPII, subunit SEC13	1.04	19.72	21.57	9.69	
309711	N/A	Hypothetical protein	1.04	34.78	30.29	16.94	

307132	30246	Alcohol dehydrogenase, class V	1.04	19.01	16.44	9.33	Probable mannitol dehydrogenase. Catalyses the NAD-dependent reduction of mannitol-1-phosphates as part of the phosphoenolpyruvate-dependent phosphotransferase system (PTS).
305158	49663	Helicase associated domain	1.03	22.51	13.03	10.79	
300961	49050	Hypothetical protein	1.03	15.55	17.22	7.52	
312017	46077	Hypothetical protein	1.02	10.02	9.07	4.89	
302791	43261	Chlorophyll A-B binding protein	1.02	32.83	27.37	16.03	
300571	22404	Pyruvate kinase	1.01	89.87	75.02	44.99	Enzyme involved in glycolysis.
308097	12902	Ferredoxin nitrite reductase	1.00	154.02	74.49	73.73	Involved in nitrogen assimilation and metabolism
311537	44098	Hypothetical protein	-1.02	82.77	144.99	158.77	
305736	34124	Hypothetical protein	-1.03	5.81	8.35	11.18	
312012	46165	Hypothetical protein	-1.03	77.75	109.37	149.10	
303162	49958	RHS Protein	-1.07	26.08	38.06	50.32	Highly conserved proteins with a broad rangeof functions, including acting as a bacterial toxin
301966	46203	Hypothetical protein	-1.07	5.06	6.79	10.05	
308224	0	Hypothetical protein	-1.07	57.44	69.15	111.84	
301896	12431	Phospholipase D1	-1.07	46.97	89.19	91.00	Linked to multiple cellular pathways, including signal transduction, membrane trafficking, and the regulation of mitosis
309445	43726	Hypothetical protein	-1.09	235.92	369.08	462.03	
307282	49933	Hypothetical protein,TP Silaffin protein	-1.10	13.39	23.29	27.00	

311237	49818	PHD-finger family protein	-1.10	24.84	35.01	48.38	Often involved in chromatin-mediated gene regulation functions, such as transcriptional coactivation.
302141	N/A	Hypothetical protein	-1.10	77.25	88.12	150.78	
305580	44194	Hypothetical protein	-1.12	5.27	8.71	10.62	
ycf88	N/A	Hypothetical protein	-1.12	1623.46	1632.03	3187.21	
302803	42977	Hypothetical protein	-1.13	23.18	34.45	46.01	
310608	14269	60S ribosomal protein L29	-1.13	193.93	244.19	391.06	
307872	51811	Porphobilinogen deaminase	-1.15	9.89	15.22	19.81	Involved in the third step of the heme biosynthetic pathway. It catalyzes the head to tail condensation of four porphobilinogen molecules into the linear hydroxymethylbilane whi le releasing four ammoniamolecules.
310565	37861	Hypothetical protein	-1.17	16.92	21.12	34.57	
305503	N/A	Hypothetical protein	-1.17	8.90	12.45	18.33	
300278	N/A	Hypothetical protein	-1.19	10.43	15.66	21.07	
306205	1766_bd	Hypothetical protein	-1.23	51.43	73.93	104.63	
306723	38777	Hypothetical protein	-1.27	5.14	8.50	11.12	
307716	44648	Hypothetical protein	-1.29	47.59	91.13	99.22	
303113	55097	Calcium-dependent protein kinase	-1.30	35.11	52.44	72.32	
301451	44100	prolin	-1.33	51.81	111.61	109.43	
307724	34125	Hypothetical protein	-1.33	10.98	20.98	23.21	
303712	11337	Translational repressor Pumilio/PUF3 and related RNA-binding proteins (Puf	-1.34	16.85	28.31	35.33	

		superfamily)					
305182	N/A	Hypothetical protein	-1.39	32.36	47.36	69.45	
301526	44000	Hypothetical protein	-1.39	13.17	18.84	28.34	
rps19	N/A	Hypothetical protein	-1.44	2947.98	2980.34	6395.22	
300122	N/A	Hypothetical protein	-1.45	6.78	8.85	15.33	
306140	54395	Fasciclin and related adhesion glycoproteins	-1.45	9.76	14.65	21.52	Conserved protein involved in cell adhesion.
310801	48558	Heat shock transcription factor	-1.51	26.77	41.29	59.67	
300097	N/A	Hypothetical protein	-1.62	48.78	72.79	111.87	
301846	N/A	DNA-dependent RNA polymerase I	-1.69	49.58	69.70	116.17	
305362	43366	Hypothetical protein	-1.71	114.81	157.59	267.73	
309485	43441	Hypothetical protein	-1.71	141.35	253.50	330.31	
308660	47898	Hypothetical protein	-1.75	9.94	15.33	23.22	
303405	51519	Lipoyltransferase or protein ligase (Biotin/lipoate A/B protein ligase family)	-1.78	47.52	81.87	113.92	Possibly creates an amide linkage that joins the free carboxyl group of a fatty acid to the epsilon-amino group of a specific lysine residue in lipoate-dependent enzymes (JGI annotation: Tony Chiovitti, 2006-02-27
300778	42600	Hypothetical protein	-1.82	50.04	107.26	120.84	
305162	16069	Dihydrolipoamide dehydrogenase, mercuric reductase	-1.84	7.71	11.40	18.51	
305254	50037	Hypothetical protein	-1.86	10.80	27.30	26.47	
301356	43312	Flavohemoprotein	-1.87	5.98	11.90	14.48	
309342	N/A	Hypothetical protein	-1.88	4.87	6.42	11.78	

302990	N/A	Hypothetical protein	-1.90	5.86	6.56	14.22	
306706	N/A	Myosin class V heavy chain or IQ calmodulin-binding motif	-1.91	4.62	7.57	11.52	
302160	46493	Glucose dehydrogenase/choline dehydrogenase/mandelonitrile lyase (GMC oxidoreductase family)	-1.92	9.00	19.37	22.11	
310699	N/A	Hypothetical protein	-1.96	7.72	13.91	19.24	
302157	46444	Hypothetical protein	-2.01	69.39	139.50	175.34	
301791	N/A	Ribonucleotide reductase, alpha subunit	-2.03	10.97	11.47	27.37	
311238	49722	sugar transporter	-2.03	9.72	21.18	25.01	
303478	0	Hypothetical protein	-2.09	9.49	13.74	24.61	
311836	11823	Core histone H2A/H2B/H3/H4	-2.17	4.05	5.84	11.22	
307519	43419	Hypothetical protein containing HMG (high mobility group) box	-2.25	3.81	6.83	10.37	
304501	47727	Hypothetical protein	-2.38	10.11	16.27	27.99	
305670	44607	Hypothetical protein	-2.38	26.18	39.98	71.64	
305079	49136	Heat shock transcription factor	-2.40	14.45	32.32	40.14	
302939	49084	FOG: Ankyrin repeat	-2.71	134.37	252.59	398.52	

309508	32875	Hypothetical protein	-2.73	5.23	12.62	15.72	
308244	N/A	Hypothetical protein	-2.79	6.57	9.86	19.52	
309152	49119	Predicted hydrolase/acyltransfe rase (alpha/beta hydrolase superfamily)	-2.80	6.76	12.13	20.33	
304615	48064	Hypothetical protein	-3.06	7.06	20.30	22.59	
308631	3388	RNA Polymerase sigma factor	-3.14	56.18	163.06	181.01	
300851	43237	Hypothetical protein	-3.16	10.59	26.16	34.63	
310736	N/A	Hypothetical protein	-3.19	12.79	20.15	41.70	
309401	50288	Hypothetical protein	-3.41	22.65	61.17	77.24	
309127	49055	Hypothetical protein	-3.44	3.35	8.53	11.26	
308074	35642	coiled-coil protein	-3.56	5.50	8.89	19.04	
302349	N/A	Hypothetical protein	-3.60	8.59	17.72	29.94	
303084	49594	Heat shock transcription factor	-4.18	48.94	171.63	192.44	Trimeric heat shock transcription factor that activates multiple genes in response to hyperthermia and recognizes variable heat shock elements (HSEs) consisting of inverted NGAAN repeats. Constitutively bound to DNA and posttranslationally regulated
304899	42608	Hypothetical protein	-4.23	17.35	57.21	68.23	
309894	N/A	Hypothetical protein	-7.62	1.65	3.55	10.71	
302577	48069	Hypothetical protein	-7.65	10.53	65.01	68.88	
307428	43365	Hypothetical protein	-15.26	2.49	34.87	35.85	

304737	48554	DNA-binding transcription factor	-19.78	27.64	327.94	575.86	Similar to mitochondrial precursor of transcription factor A (mtTFA), and contains a high mobility group (HMG) box with DNA binding activity
