# Supplementary material for: Genetic Manipulation of Competition for Nitrate between Heterotrophic Bacteria and Diatoms
Source: Front Microbiol. 2016 Jun 9;7:880. doi: 10.3389/fmicb.2016.00880 (PMC4899447; doi:10.3389/fmicb.2016.00880)
Supplement: Supplementary file 5 [file Table5.PDF]

Supplementary Table 5. Putative *P. tricornutum* genes expressed during the exponential and stationary sampling points that are potentially involved in NH_4^- transport and utilization, including gene IDs, annotation, average reads per kilobase of transcript per million mapped reads (RPKM) for each treatment (*P. tricornutum* monoculture, *P. tricornutum-A. macleodii* WT co-culture, and *P. tricornutum-A. macleodii* Δ*nasA* co-culture), and standard deviation of n = 3 replicate cultures.

		Exponential Sampling Point (Day 5)						Stationary Sampling Point (Day 13)					
Gene ID (Phatr3)	**Putative Gene**	*P. tricornutum* Only (RPKM)	St. Dev	*P. tricornutum + A. macleodii* WT	St. Dev	*P. tricornutum + A. macleodii* Δ*nasA*	St. Dev	*P. tricornutum* Only (RPKM)	St. Dev	*P. tricornutum + A. macleodii* WT	St. Dev	*P. tricornutum + A. macleodii* Δ*nasA*	St. Dev
302073	L-serine ammonia-lyase	30.65	3.12	30.20	5.49	30.39	7.85	24.14	3.18	16.84	3.88	21.91	3.02
306207	Ammonium_transporter	1.45	0.46	1.05	1.17	1.08	0.41	0.91	0.11	0.84	0.64	0.73	0.11
304169	Predicted protein Fragment	10.41	6.85	4.99	3.50	6.56	1.04	8.01	1.68	5.44	1.96	6.79	0.87
310330	Ammonia permease	11.23	2.20	8.97	1.09	8.40	3.32	21.97	2.37	17.12	3.31	20.68	3.50
306230	Ammonia permease	878.62	376.79	1067.70	814.11	897.49	568.63	4887.57	339.65	5515.14	64.30	4847.09	116.81
308405	Ammonia permease	58.44	15.16	52.37	18.07	51.67	13.23	117.75	5.40	93.70	20.01	98.46	6.11
309469	Ammonia permease	3.58	1.37	5.72	1.07	4.50	1.16	14.41	3.09	15.63	5.26	11.33	2.09
309517	Ammonium transporter RHBG	32.97	27.94	36.09	51.84	27.84	36.26	103.04	14.30	63.17	21.91	101.41	21.09
311079	Ammonia permease	3.42	1.53	1.59	1.16	2.83	1.10	2.30	0.29	1.65	0.25	1.70	0.38
311078	L-threonine ammonia-lyase	9.99	1.17	12.62	2.52	13.23	3.64	14.81	1.75	15.49	2.73	14.43	0.84
301095	L-serine ammonia-lyase Fragment (EC 4.3.1.17)	32.72	15.92	37.54	17.63	41.15	21.96	57.84	7.17	42.05	3.37	50.50	4.98
307308	CTP synthase (UTP-ammonia lyase)	19.70	26.28	28.27	18.00	32.11	20.96	47.34	1.07	40.89	5.99	45.28	5.05
309585	CPS III, carbamoyl-phosphate synthase mitochindrial	49.59	23.68	64.51	19.68	66.21	27.54	30.66	3.40	26.60	3.75	28.85	5.02
309818	Ammonia permease	5.43	2.44	5.73	1.36	7.64	1.16	8.63	2.50	6.50	0.68	7.38	1.47
303643	Aspartate--ammonia ligase	28.19	3.11	22.66	9.24	27.17	5.38	69.70	5.50	64.82	2.45	67.87	2.89
307824	aspartate--ammonia ligase	80.84	19.19	51.90	14.22	65.31	24.93	151.57	6.79	162.62	7.31	152.48	12.07
303699	Ammonia permease	0.00	0.00	0.24	0.04	0.00	0.00	0.17	0.16	0.18	0.16	0.03	0.03
