# Supplementary material for: Genetic Manipulation of Competition for Nitrate between Heterotrophic Bacteria and Diatoms
Source: Front Microbiol. 2016 Jun 9;7:880. doi: 10.3389/fmicb.2016.00880 (PMC4899447; doi:10.3389/fmicb.2016.00880)
Supplement: Supplementary file 6 [file Table6.PDF]

Supplementary Table 6. Average specific growth rate (μ) and standard deviation (St. Dev.) of *P. tricornutum* – *A. macleodii* WT co-cultures during 5 rounds of semi-continuous culture transfer.

Transfer Round	Growth Rate (μ)	St. Dev.
1	1.62	0.17
2	1.51	0.20
3	1.33	0.05
4	1.25	0.17
5	1.37	0.09
