# Supplementary material for: Genetic Manipulation of Competition for Nitrate between Heterotrophic Bacteria and Diatoms
Source: Front Microbiol. 2016 Jun 9;7:880. doi: 10.3389/fmicb.2016.00880 (PMC4899447; doi:10.3389/fmicb.2016.00880)
Supplement: Supplementary file 7 [file Image1.PDF]

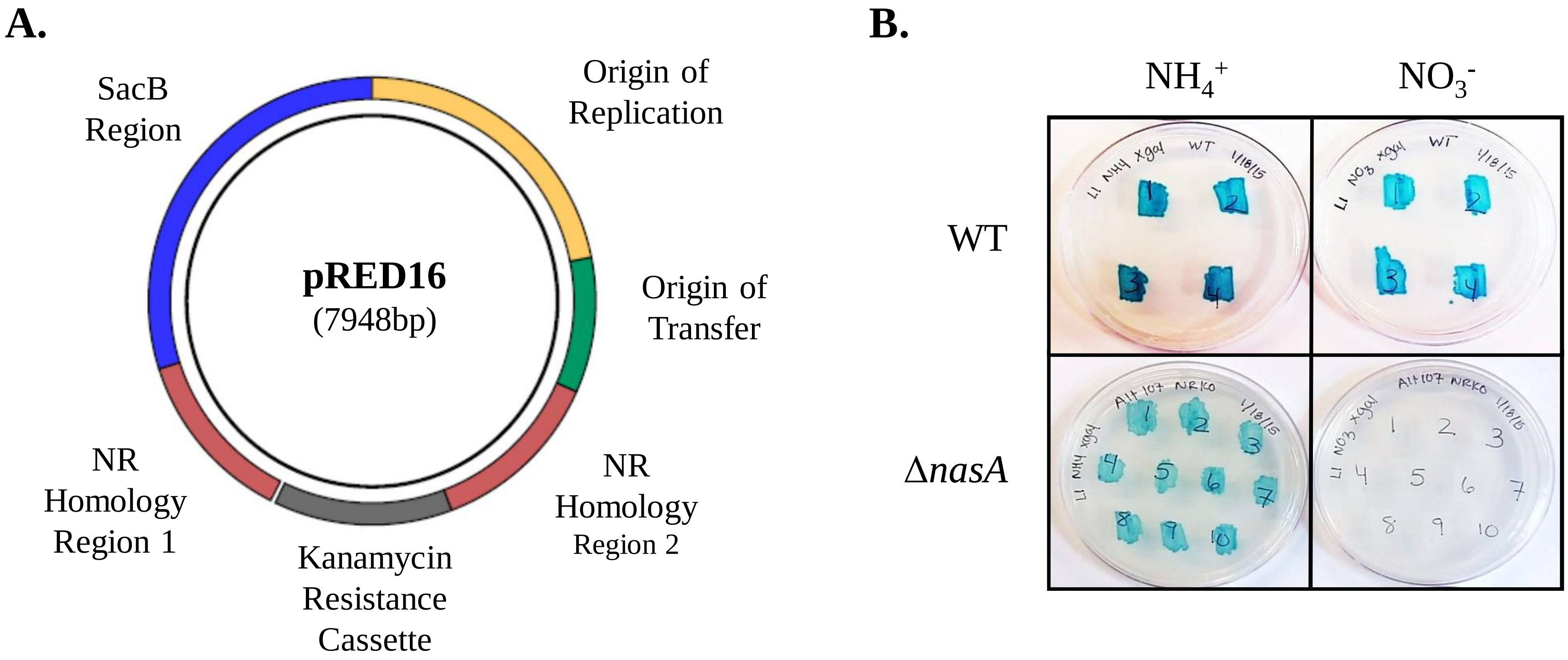

Supplementary Figure 1: Tools used to engineer and confirm the Δ*nasA* *A. macleodii* strain. (A) The plasmid pRED16, which contains: an origin of replication (from source plasmid pBBR1MCS-5, an origin of transfer (from source plasmid pRL2948a), two regions with homology to the *A. macleodii* NR gene (amplified from *A. macleodii* genomic DNA) flanking a kanamycin resistance cassette, and the SacB gene (from source plasmid pRL2948a). (B) confirmation of the NR knockout phenotype; WT *A. macleodii* can grow on X-gal ASW/Agar plates using either NO_3^- or NH_4^+ as a nitrogen source, while the Δ*nasA* strain can only grow using NH_4^+ as a nitrogen source, with growth visualized by the blue appearance of colonies.
