# Supplementary material for: Genetic Manipulation of Competition for Nitrate between Heterotrophic Bacteria and Diatoms
Source: Front Microbiol. 2016 Jun 9;7:880. doi: 10.3389/fmicb.2016.00880 (PMC4899447; doi:10.3389/fmicb.2016.00880)
Supplement: Supplementary file 8 [file Image2.PDF]

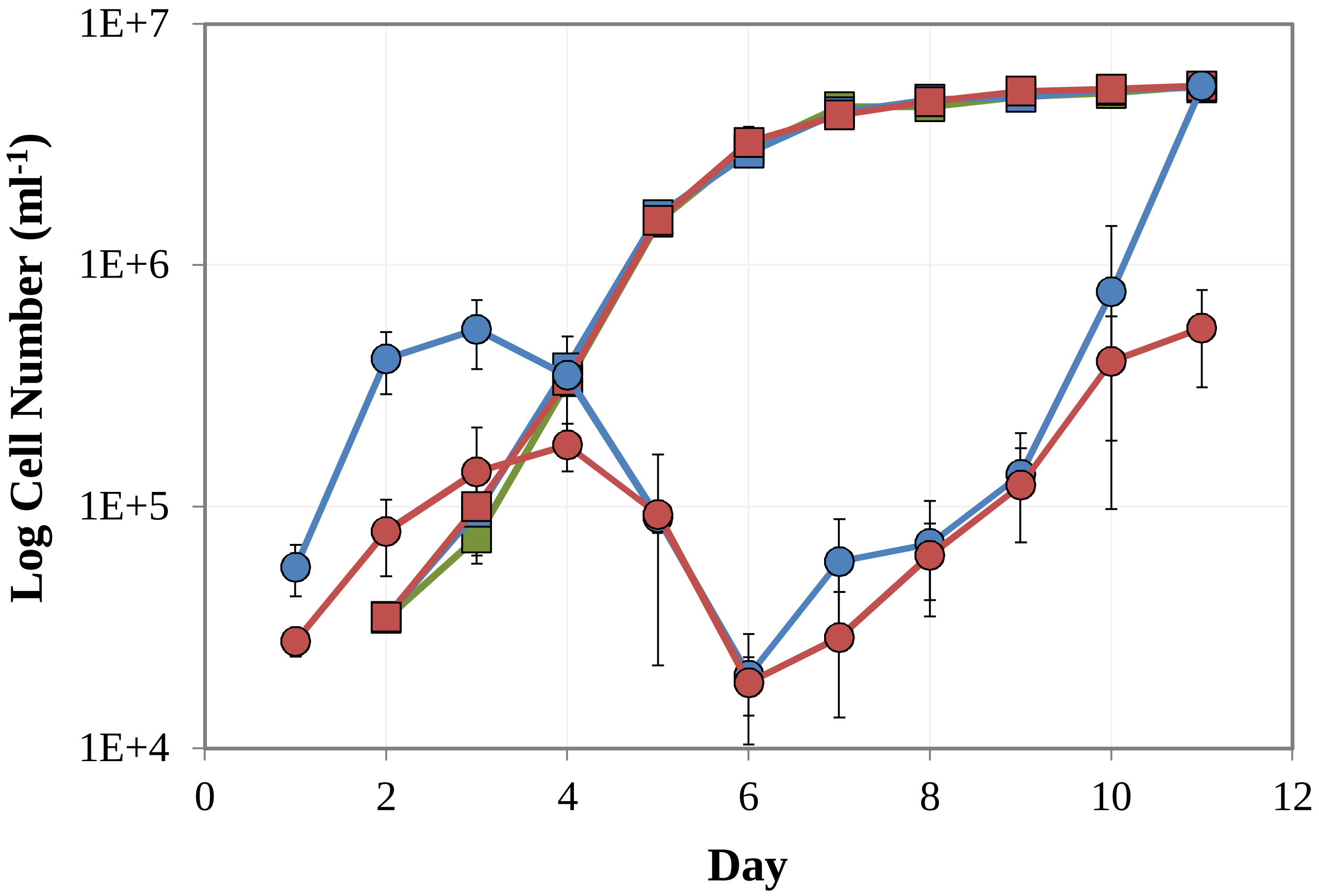

Supplementary Figure 2: Log cell numbers of diatoms and bacteria in the baseline experiment as determined by manual cell counts. Squares = diatom cell numbers and circles = bacterial cell numbers. Green markers = *P. tricornutum* monocultures, blue markers = *P. tricornutum- A. macleodii* WT co-cultures, and red markers = *P. tricornutum- A. macleodii* Δ*nasA* co-cultures
