# Supplementary material for: Genetic Manipulation of Competition for Nitrate between Heterotrophic Bacteria and Diatoms
Source: Front Microbiol. 2016 Jun 9;7:880. doi: 10.3389/fmicb.2016.00880 (PMC4899447; doi:10.3389/fmicb.2016.00880)
Supplement: Supplementary file 9 [file Image3.PDF]

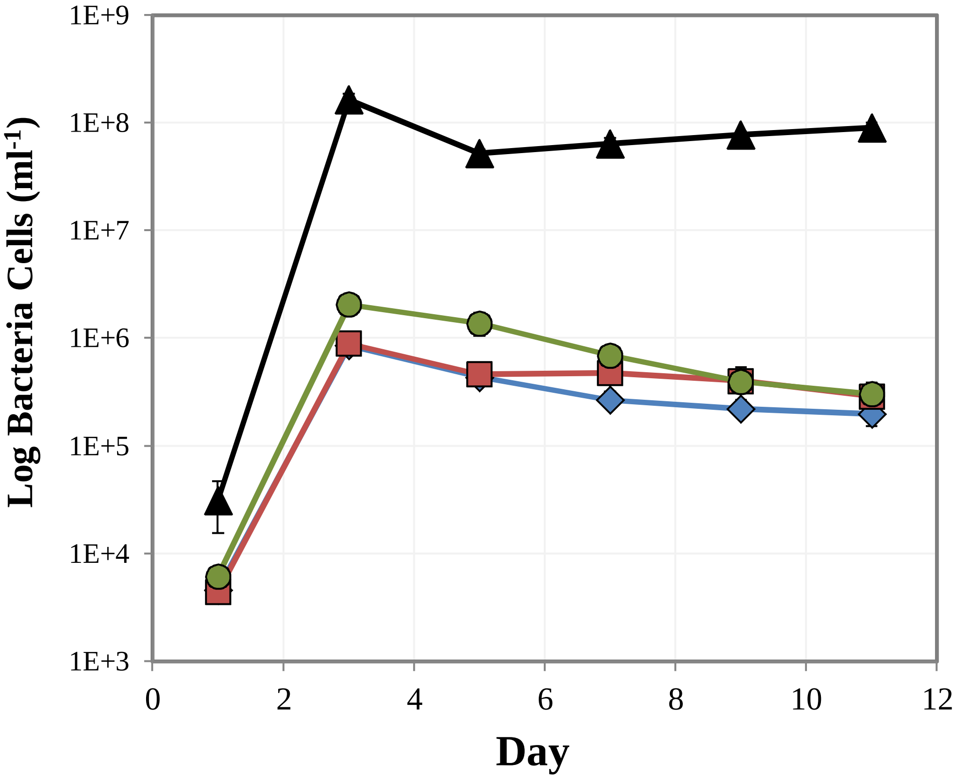

Supplementary Figure 3: Log number of *A. macleodii* WT when grown on different types of media. Black triangles= marine broth (MB) media, green circles= *P. tricornutum* filtrate media, blue diamonds = Aquil ASW with NO_3^- added to a final concentration of 300 µM, and red squares = Aquil ASW with no nitrogen source added. N = 3 replicates, and error bars are standard deviation.
