# Supplementary material for: Genetic Manipulation of Competition for Nitrate between Heterotrophic Bacteria and Diatoms
Source: Front Microbiol. 2016 Jun 9;7:880. doi: 10.3389/fmicb.2016.00880 (PMC4899447; doi:10.3389/fmicb.2016.00880)
Supplement: Supplementary file 10 [file Image4.PDF]

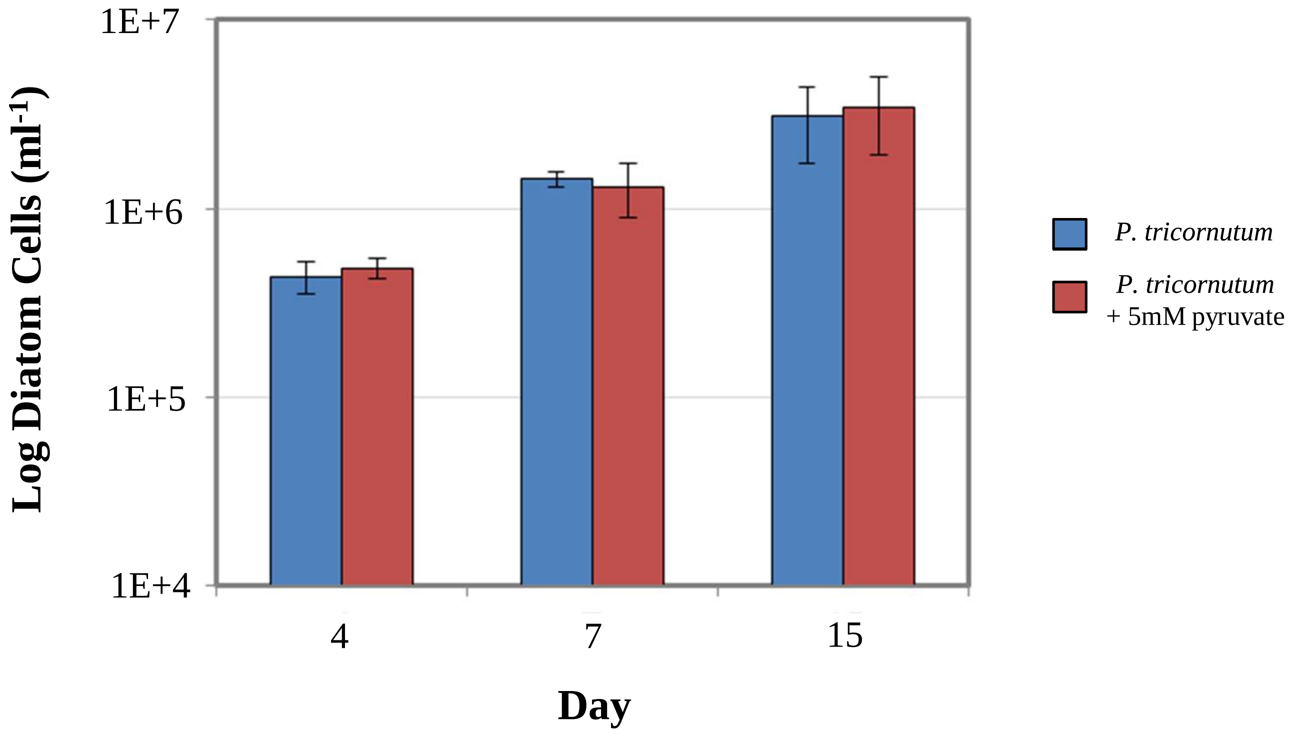

Supplementary Figure 4: Log cell numbers of WT *P. tricornutum* grown with (red bars) and without (blue bars) the addition of pyruvate as a DOC source to a 5 mM final concentration. Cell counts were determined via flow cytometry on days 4, 7, and 15 of the experiment. N = 3 replicates, and error bars are standard deviation.
