# Supplementary material for: Genetic Manipulation of Competition for Nitrate between Heterotrophic Bacteria and Diatoms
Source: Front Microbiol. 2016 Jun 9;7:880. doi: 10.3389/fmicb.2016.00880 (PMC4899447; doi:10.3389/fmicb.2016.00880)
Supplement: Supplementary file 11 [file Image5.PDF]

Supplementary Table 5. Putative *P. tricornutum* genes expressed during the exponential and stationary sampling points that are potentially involved in $NH_4^-$ transport and utilization, including gene IDs, annotation, average reads per kilobase of transcript per million mapped reads (RPKM) for each treatment (*P. tricornutum* monoculture, *P. tricornutum-A. macleodii* WT co-culture, and *P. tricornutum-A. macleodii* Δ*nasA* co-culture), and standard deviation of n = 3 replicate cultures.

| | | Exponential Sampling Point | | | | | | Stationary Sampling Point | | | | | |
|---|---|---|---|---|---|---|---|---|---|---|---|---|---|
| Gene ID (Phatr3) | **Putative Gene** | *P. tricornutum* Only (RPKM) | St. Dev | *P. tricornutum + A. macleodii* WT | St. Dev | *P. tricornutum + A. macleodii* Δ*nasA* | St. Dev | *P. tricornutum* Only (RPKM) | St. Dev | *P. tricornutum + A. macleodii* WT | St. Dev | *P. tricornutum + A. macleodii* Δ*nasA* | St. Dev |
| 302073 | L-serine ammonia-lyase | 30.65 | 3.12 | 30.20 | 5.49 | 30.39 | 7.85 | 24.14 | 3.18 | 16.84 | 3.88 | 21.91 | 3.02 |
| 306207 | Ammonium_transporter | 1.45 | 0.46 | 1.05 | 1.17 | 1.08 | 0.41 | 0.91 | 0.11 | 0.84 | 0.64 | 0.73 | 0.11 |
| 304169 | Predicted protein Fragment | 10.41 | 6.85 | 4.99 | 3.50 | 6.56 | 1.04 | 8.01 | 1.68 | 5.44 | 1.96 | 6.79 | 0.87 |
| 310330 | Ammonia permease | 11.23 | 2.20 | 8.97 | 1.09 | 8.40 | 3.32 | 21.97 | 2.37 | 17.12 | 3.31 | 20.68 | 3.50 |
| 306230 | Ammonia permease | 878.62 | 376.79 | 1067.70 | 814.11 | 897.49 | 568.63 | 4887.57 | 339.65 | 5515.14 | 64.30 | 4847.09 | 116.81 |
| 308405 | Ammonia permease | 58.44 | 15.16 | 52.37 | 18.07 | 51.67 | 13.23 | 117.75 | 5.40 | 93.70 | 20.01 | 98.46 | 6.11 |
| 309469 | Ammonia permease | 3.58 | 1.37 | 5.72 | 1.07 | 4.50 | 1.16 | 14.41 | 3.09 | 15.63 | 5.26 | 11.33 | 2.09 |
| 309517 | Ammonium transporter RHBG | 32.97 | 27.94 | 36.09 | 51.84 | 27.84 | 36.26 | 103.04 | 14.30 | 63.17 | 21.91 | 101.41 | 21.09 |
| 311079 | Ammonia permease | 3.42 | 1.53 | 1.59 | 1.16 | 2.83 | 1.10 | 2.30 | 0.29 | 1.65 | 0.25 | 1.70 | 0.38 |
| 311078 | L-threonine ammonia-lyase | 9.99 | 1.17 | 12.62 | 2.52 | 13.23 | 3.64 | 14.81 | 1.75 | 15.49 | 2.73 | 14.43 | 0.84 |
| 301095 | L-serine ammonia-lyase Fragment (EC 4.3.1.17) | 32.72 | 15.92 | 37.54 | 17.63 | 41.15 | 21.96 | 57.84 | 7.17 | 42.05 | 3.37 | 50.50 | 4.98 |
| 307308 | CTP synthase (UTP-ammonia lyase) | 19.70 | 26.28 | 28.27 | 18.00 | 32.11 | 20.96 | 47.34 | 1.07 | 40.89 | 5.99 | 45.28 | 5.05 |
| 309585 | CPS III, carbamoyl-phosphate synthase mitochindrial | 49.59 | 23.68 | 64.51 | 19.68 | 66.21 | 27.54 | 30.66 | 3.40 | 26.60 | 3.75 | 28.85 | 5.02 |
| 309818 | Ammonia permease | 5.43 | 2.44 | 5.73 | 1.36 | 7.64 | 1.16 | 8.63 | 2.50 | 6.50 | 0.68 | 7.38 | 1.47 |
| 303643 | Aspartate--ammonia ligase | 28.19 | 3.11 | 22.66 | 9.24 | 27.17 | 5.38 | 69.70 | 5.50 | 64.82 | 2.45 | 67.87 | 2.89 |
| 307824 | aspartate--ammonia ligase | 80.84 | 19.19 | 51.90 | 14.22 | 65.31 | 24.93 | 151.57 | 6.79 | 162.62 | 7.31 | 152.48 | 12.07 |
| 303699 | Ammonia permease | 0.00 | 0.00 | 0.24 | 0.04 | 0.00 | 0.00 | 0.17 | 0.16 | 0.18 | 0.16 | 0.03 | 0.03 |
